# TCMToxDB: a comprehensive database for the toxicological analysis of traditional Chinese medicines

**DOI:** 10.1093/database/baag019

**Published:** 2026-04-16

**Authors:** Yongzheng Zhu, Laihao Fang, Yunbo Miao, Longfei Ma, Rong Sun, Yimin Mao, Wei Guo, Jun Wang, Guoxian Yu

**Affiliations:** School of Software, Shandong University, 1500 Shunhua Road, 250101, Shandong, China; SDU-NTU Centre for Artificial Intelligence Research, Shandong University, 1500 Shunhua Road, 250101, Shandong, China; School of Software, Shandong University, 1500 Shunhua Road, 250101, Shandong, China; SDU-NTU Centre for Artificial Intelligence Research, Shandong University, 1500 Shunhua Road, 250101, Shandong, China; School of Software, Shandong University, 1500 Shunhua Road, 250101, Shandong, China; SDU-NTU Centre for Artificial Intelligence Research, Shandong University, 1500 Shunhua Road, 250101, Shandong, China; School of Software, Shandong University, 1500 Shunhua Road, 250101, Shandong, China; SDU-NTU Centre for Artificial Intelligence Research, Shandong University, 1500 Shunhua Road, 250101, Shandong, China; The Second Hospital of Shandong University, Shandong University, 247 North Park Street, 250000, Shandong, China; Renji Hospital, Shanghai Jiao Tong University, 160 Pujian Road, 200127, Shanghai, China; SDU-NTU Centre for Artificial Intelligence Research, Shandong University, 1500 Shunhua Road, 250101, Shandong, China; SDU-NTU Centre for Artificial Intelligence Research, Shandong University, 1500 Shunhua Road, 250101, Shandong, China; School of Software, Shandong University, 1500 Shunhua Road, 250101, Shandong, China; SDU-NTU Centre for Artificial Intelligence Research, Shandong University, 1500 Shunhua Road, 250101, Shandong, China

## Abstract

The safety and modernization of traditional Chinese medicine (TCM) are significant concerns for human beings. Recently, the adverse effects caused by the use of certain TCMs have been frequently reported. Although TCMs may cause toxic reactions, they also play critical roles in treating multiple complex diseases. Therefore, toxicity research is urgently needed for the safe usage of TCMs. However, existing databases for TCMs primarily focus on the pharmacological effects of TCMs, with limited attention to the toxicity. They neither distinguish the toxic effects of formulas, herbs, and ingredients, nor classify and summarize targets for specific toxic manifestations, or assemble evidence from previous studies. We developed TCMToxDB, a comprehensive database that focuses on the toxicity and safe usage of TCMs. TCMToxDB systematically integrates and analyses the research results of toxic TCMs, offering users diverse information acquisition and analysis services. In addition, it assembles five canonical herb–target and ingredient–target interaction prediction algorithms with different advantages, which support the prediction of toxic targets of herbs and ingredients to empower the toxicity research of TCMs and to meet users’ personal needs. TCMToxDB is accessible at https://www.sdu-idea.cn/TCMToxDB.

**Database URL:**  https://www.sdu-idea.cn/TCMToxDB

## Introduction

Traditional Chinese medicine (TCM), as a treasure of ancient Chinese science, boasts a long history and extensive clinical applications. Over thousands of years, TCM has accumulated a rich clinical experience and theoretical system, which has not only made significant contributions to the health of the Chinese people, but also played a unique role in the health and well-being of people around the world [1]. In recent years, with the deepening research in TCM and the rapid advancement of modern science and technology, the research and application of TCM have embraced new opportunities [2]. The therapeutic concepts and efficacy of TCM are gradually gaining international recognition, and more research institutions are now dedicating to TCM research [3]. However, the complexity and diversity of TCMs also bring numerous challenges, particularly in the study of toxicity in TCM, which has become a crucial issue in ensuring its safety and efficacy [4]. Some herbs or ingredients may cause adverse reactions or even severe toxic events when used improperly, in excessive doses, or over long periods [5]. For example, herb *Xi Xin* and ingredient *aristolochic acid* have drawn significant attention due to their potential toxicity [6]. These issues restrict the clinical use of TCM and hinder its internationalization and modernization.

Databases play an important role in the field of TCM research [7, 8]. The databases commonly used in TCM research can be divided into the following categories. (i) Disease-related databases collect information on TCMs and their ingredients (similar to chemical drugs) regarding their therapeutic effects on specific diseases, as well as the associated liver injuries. These databases provide valuable references for the personalized treatment of specific diseases and help to optimize the clinical application of TCMs. Examples include LiverTox [9], Hepatox [10], and others [11, 12]. (ii) Ingredient-related databases collect information related to ingredients of TCMs, including the active ingredients, chemical structures, and physicochemical properties. Such data provide a critical foundation for the research of pharmacologically active substances of TCMs. Such as TCMID [13], HERB [14], and TCMSP [15]. (iii) Mechanism and target-related databases collect information on molecules and their targets, offering valuable resources for exploring the functions and mechanisms of various ingredients in TCMs. Examples include CTD [16], SIDER [17], ETCM [18], and TCMBank [19]. (iv) Metabolism-related databases provide information on the toxic metabolism of chemical drugs. Since certain ingredients of TCMs can be also regarded as chemical drugs, this type of database serves as a foundational resource for toxicological and metabolic studies of TCMs. For example, HIM [20], HMDB [21], and METLIN [22]. (v) Risk assessment-related databases compile information on potentially toxic chemical elements and utilize simulation models to conduct risk assessments, thereby improving the risk evaluation. Such data and models can be applied to assess the risk of toxic ingredients in TCMs. Such as MRTCM [23] and TOXNET [24].

In toxicity research within TCM, several authoritative biomedical databases are utilized. For instance, CTD [16] primarily focuses on chemical drug research, excels in elucidating the toxicological mechanisms of chemical drugs, but its coverage of toxicity-related targets and pathways is limited to specific TCMs. SIDER [17] establishes a standardized terminology system for adverse drug reactions; however, it lacks data on the unique toxicological profiles of TCMs. In the field of hepatotoxicity, LiverTox [9] focuses on integrating clinical evidence of liver injury caused by chemical drugs. Hepatox [10] includes cases related to TCMs, but it lacks robust causal evidence and covers only a limited number of TCMs. Meanwhile, a series of databases specifically tailored for TCM has also been developed within the field. TCMID [13], HERB [14], TCMSP [15], ETCM [18], and TCMKD [25] focus on ingredient–target network pharmacology analyses. However, their integration of toxicity-related data for TCMs is limited to simply noting the presence or absence of toxicity, lacking in-depth analysis. HIM [20] utilizes metabolomics technologies to innovatively study the toxicity of chemical drugs, but its research scope is limited and lacks a systematic evaluation of the toxicity of TCMs.

In summary, the current databases used for research on toxicity in TCM mainly focus on pharmacology and ingredient–target analysis. However, their research on toxicity in TCM lacks comprehensive and systematic evaluation, and sufficient investigation into the multi-dimensional complex relationships between toxic ingredients and their biological targets. Therefore, it is urgent to develop a holistic TCM toxicity database to safeguard the usage of TCMs and facilitate the toxic research of TCMs. This database should systematically integrate toxic TCM research data and provide comprehensive analysis functions.

To address the above issues, we developed the TCMToxDB, a comprehensive database that focuses on the safe use of TCMs. TCMToxDB systematically integrates and analyses the research results of toxic TCMs, and provides the functions of toxic target prediction of TCMs and of ingredients to empower toxicity TCM research. There are two main characteristics of TCMToxDB: (i) a wide range of properties and toxicity studies of formulas, herbs, ingredients, and targets are collected and curated, and all are supported by the biomedical literature. At the same time, it provides authentic analytical functions, empowering systematic exploration of toxicological interactions and relationships among formulas, herbs, ingredients, and targets. (ii) It provides an automatic workflow for predicting the toxicity targets of herbs and ingredients, which integrates five widely used algorithms [26–30] with different advantages to meet users’ personalized needs.

## Materials and methods

### Framework of TCMToxDB

TCMToxDB is dedicated to providing professionals and practitioners in the fields of traditional medicine, pharmacology, toxicology, and biomedical research with an in-depth and comprehensive understanding of toxicity in TCM. It aggregates the toxicity information of formulas, herbs, ingredients, and targets, and offers a promising visualization and analysis platform for accessing the toxicity data of TCM. In addition, it provides a toxicity target prediction tool that enables users to predict the toxicity targets of herbs and ingredients.

**Figure 1:**
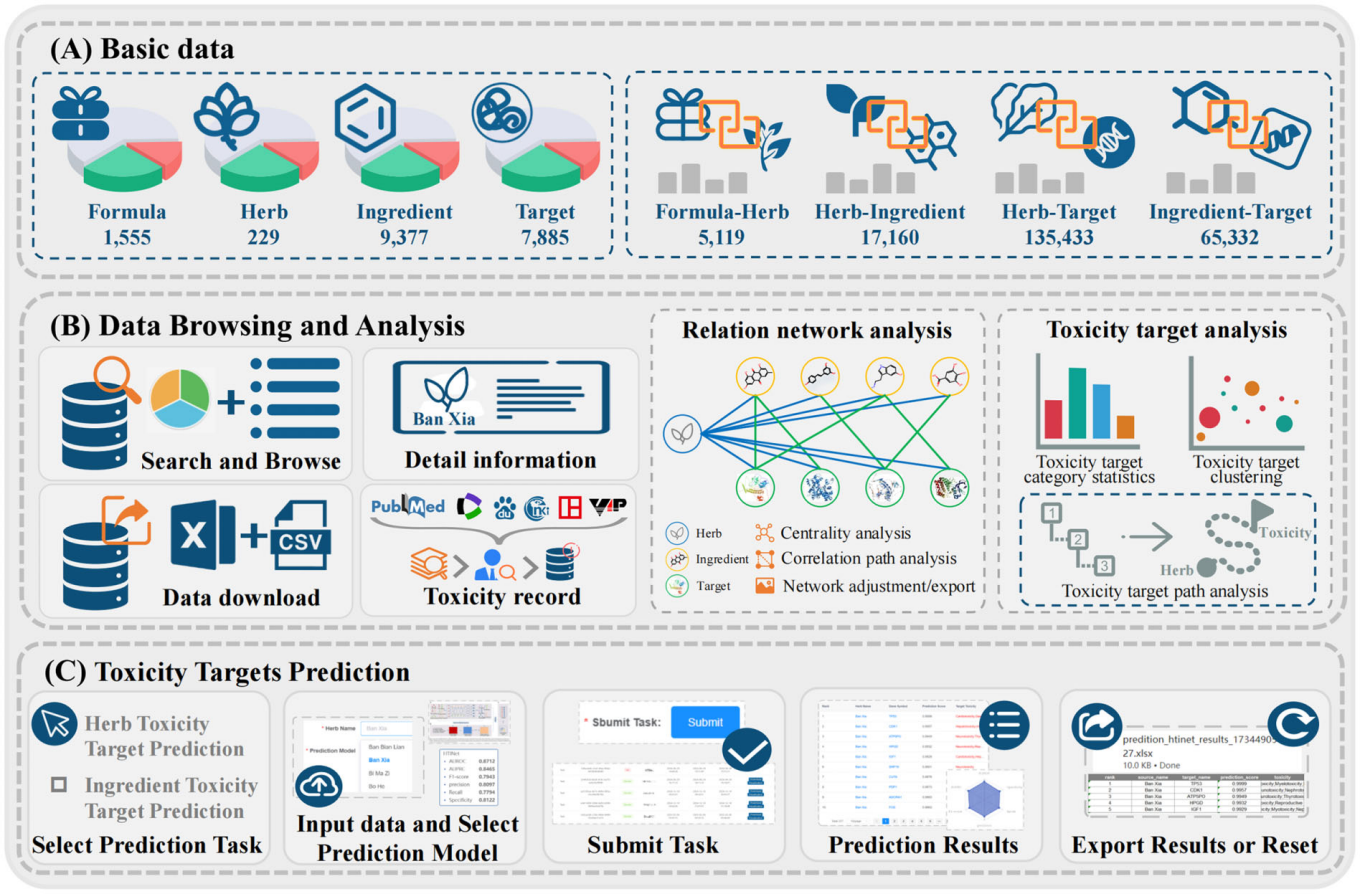
Framework overview of TCMToxDB. (A) The basic data of TCMToxDB include basic information of formula, herbs, ingredients, targets, formula–herb interactions, herb–ingredient interactions, herb–target interactions, and ingredient–target interactions, the data storage is powered by MySQL. (B) Data browse and analysis comprises six parts: data search and browse, download, detail information, toxicity record, relation network analysis, and toxicity target analysis. (C) Toxicity target prediction: TCMToxDB provides computational tools with automatic workflow for predicting toxicity targets of herbs and ingredients.

As shown in Fig. 1, TCMToxDB integrates eight resources for TCM toxicity analysis: basic information of formula, herbs, ingredients, targets, and formula–herb interactions, herb–ingredient interactions, herb–target interactions, and ingredient–target interactions. Table 1 gives the sources of these data. The various data of TCMToxDB will be maintained through a pipeline program, which can update and expand all content. We will maintain TCMToxDB to keep data updated and provide users with trustable services.

**Table 1:** Data sources of TCMToxDB.

Data type	Size	Source	Link
Formula	1555	ChP (2020) [31]	https://ydz.chp.org.cn/#/main
		ETCM 2.0 [18]	http://www.tcmip.cn/ETCM2/front/#/
Herb	229	ChP (2020) [31]	https://ydz.chp.org.cn/#/main
		HERB [14]	http://herb.ac.cn/
		TCMID 2.0 [13]	http://www.megabionet.org/tcmid/
		TCM-ID [32]	https://www.bidd.group/TCMID/
		TCMSP [15]	https://old.tcmsp-e.com/tcmsp.php
		SymMap v2 [33]	http://www.symmap.org/
		TCMBank [19]	https://tcmbank.cn/
		HIT 2.0 [34]	http://www.badd-cao.net:2345/
Ingredient	9377	HERB [14]	http://herb.ac.cn/
		TCMID 2.0 [13]	http://www.megabionet.org/tcmid/
		TCM-ID [32]	https://www.bidd.group/TCMID/
		TCMSP [15]	https://old.tcmsp-e.com/tcmsp.php
		SymMap v2 [33]	http://www.symmap.org/
		CAS [35]	https://www.cas.org/zh-hans/cas-data
		PubChem [36]	https://pubchem.ncbi.nlm.nih.gov/
		DrugBank [37]	https://www.drugbank.com/
Target	7885	TTD [38]	https://db.idrblab.net/ttd/
		NCBI [39]	https://www.ncbi.nlm.nih.gov/
		UniProt [40]	https://www.uniprot.org/
		GeneCards [41]	https://www.genecards.org
Formula–herb Interaction	5119	ChP (2020) [31]	https://ydz.chp.org.cn/#/main
		ETCM 2.0 [18]	http://www.tcmip.cn/ETCM2/front/#/
Herb–ingredient Interaction	16603	SymMap v2 [33]	http://www.symmap.org/
		HERB [14]	http://herb.ac.cn/
		TCMBank [19]	https://tcmbank.cn/
		HIT 2.0 [34]	http://www.badd-cao.net:2345/
Herb–target Interaction	135433	SymMap v2 [33]	http://www.symmap.org/
		HERB [14]	http://herb.ac.cn/
		TCMBank [19]	https://tcmbank.cn/
		HIT 2.0 [34]	http://www.badd-cao.net:2345/
Ingredient–target Interaction	65332	SymMap v2 [33]	http://www.symmap.org/
		HERB [14]	http://herb.ac.cn/
		TCMBank [19]	https://tcmbank.cn/
		HIT 2.0 [34]	http://www.badd-cao.net:2345/
Toxicity reference	1134	Google Scholar	https://scholar.google.com
		CNKI	https://www.cnki.net/
		VIP	https://www.cqvip.com/
		WanFang	https://www.wanfangdata.com.cn/index.html
		PubMed [42]	https://pubmed.ncbi.nlm.nih.gov/
		WOS	https://www.webofscience.com/wos/

The data browsing module is composed of six parts: (i) information search and browse, (ii) download, (iii) detail information, (iv) toxicity record, (v) relation network analysis, and (vi) toxicity target analysis. On the other hand, the toxicity target prediction tool is built on a workflow with five steps: (i) select prediction task, (ii) input data and select prediction model, (iii) submit task, (iv) results visualization, and (v) results export or reset.

### Data sources and collection

As shown in Table 1, TCMToxDB currently includes 1555 formulas (217 toxic formulas), 229 toxic herbs, 9377 ingredients, 7885 targets (2047 toxic targets), 5119 formula–herb interactions, 135 433 herb–target interactions, 16 603 herb–ingredient interactions, 65 332 ingredient–target interactions, and 1134 toxicity references (formulas and herbs). It is worth noting that the toxic information was specifically identified and curated through a combination of external database mining and comprehensive literature review, rather than being pre-labelled in the source databases.

The formulas included in TCMToxDB were obtained from two databases: ChP (2020) [31] and ETCM 2.0 [18]. We comprehensively recorded each formula’s names (e.g. Chinese name, PinYin name), syndrome, dosage form, formula type, prescription, related diseases, indications, efficacy, and formula–herb association. We carefully carried out the data cleaning process for formulas to ensure accuracy and consistency. First, we standardized all formula names based on the national standard GB/T 31773-2015, unifying different names referring to the same formula. We corrected errors in dosage forms, syndrome types, and efficacy descriptions by comparing the two databases and checking against the standard. Then, we identified and merged duplicate entries to keep all unique information while removing repeats. We also carefully checked the herb ingredients in each formula and supplemented or corrected missing or conflicting information about diseases and uses. It is noteworthy that information regarding formula’s toxicity was obtained through literature retrieval, and the specific collection process will be described at the end of this section.

In TCMToxDB, we integrated 229 toxicity herbs’ information from the ChP (2020) [31], HERB [14], TCMID 2.0 [13], TCM-ID [32], TCMSP [15], SymMap v2 [33], TCMBank [19], and HIT 2.0 [34] databases, including their names (e.g. Chinese name, English name, Latin name), properties (e.g. cold or warm, bitter or acrid or sweet), meridians (e.g. liver, lung or other channels), used parts (e.g. root, stem, leaf, flower or seed), functions, indications, toxicity, clinical manifestations, therapeutic, etc. Besides, Chinese names, Pinyin names, and English names of herbs were normalized. Details of toxic herbs data cleaning are as follows. First, all Chinese herb names were converted to simplified Chinese characters to ensure character-level consistency. Second, polyphonic characters in Chinese names were identified, and their standard pronunciations were confirmed according to authoritative references. Third, homophones were clearly distinguished and annotated. Next, information pertaining to the same herb from different sources was systematically merged; conflicting entries (e.g. discrepant meridian tropisms or toxicity descriptions) were resolved by prioritizing the Chinese Pharmacopoeia (2020) and the national standard GB/T 31774-2015. Redundant or duplicate records were identified and removed to form a non-repetitive, structured dataset. It is noteworthy that information regarding herbal toxicity was obtained through literature retrieval, and the specific collection process will be described at the end of this section.

The ingredients of herbs included in TCMToxDB were mainly from database integration and literature mining. First, ingredients were systematically collected from databases, including HERB [14], TCMID 2.0 [13], TCM-ID [32], TCMSP [15], and SymMap v2 [33]. For each ingredient, information such as name, molecular SMILES, molecular formula, molecular weight, association with other databases, ingredient–herb association, and ingredient–target association is provided. We normalized the ingredients as follows: mapping to the PubChem ID by PubChemPy package, labelling with the chemical name, chemical formula, CAS number, PubChem CID, and DrugBank number by semi-automatic programming and manual checking, and filling the missing value with NA. As a result, 9377 ingredients were finally incorporated into TCMToxDB.

The targets of formulas and herbs recorded in TCMToxDB were manually collected and organized according to the TTD [38], NCBI (NCBI Protein database) [39], Uniprot [40], and GeneCards [41]. The basic information includes the name, gene symbol, dosage form, target type, chromosome, map location, sequence, association with other databases, target–herb association, and target–ingredient association. In the target name standardization process, all targets’ names (target name, gene symbol, UniProt ID, and DrugBank ID) were normalized by R package GeneBook (https://CRAN.R-project.org/package=GeneBook). Toxicity-related information for these targets was retrieved from the GeneCards database using keywords such as ‘toxicity’, ‘adverse effect’, and ‘side effect’, focusing on gene–disease associations, pathological pathways, and relevant pharmacological annotations. The retrieved data were then manually reviewed to ensure biological relevance and accuracy in the context of TCM.

For the formula–herb association, data were collected and organized from databases ChP (2020) [31] and ETCM 2.0 [18]. For the herb–ingredient, herb–target, and ingredient–target associations, data were gathered and curated from databases SymMap v2 [33], HERB [14], TCMBank [19], and HIT 2.0 [34].

For the toxicity information of formulas and herbs, along with supporting literature, data were initially collected through targeted web crawling using Python-based automated scripts from multiple academic databases, including Google Scholar (https://scholar.google.com), CNKI (China National Knowledge Infrastructure, https://www.cnki.net/), VIP (China Science and Technology Journal Database, https://www.cqvip.com/), WanFang (Wanfang Data, https://www.wanfangdata.com.cn/index.html), PubMed (https://pubmed.ncbi.nlm.nih.gov/), and WOS (Web of Science, https://www.webofscience.com/wos/). During retrieval, we simultaneously recorded bibliographic metadata (title, year, journal, DOI/PMID when available, and source database) to facilitate traceability and subsequent de-duplication; duplicate records were merged primarily by DOI/PMID and, when identifiers were missing, by normalized title matching with manual confirmation for ambiguous cases. We developed a multilingual keyword system incorporating Chinese, English, Latin scientific names, and common synonyms for each herb and formula (such as ‘Bu Gu Zhi toxicity’, ‘Malaytea Scurfpea toxicity’, and ‘Psoralea corylifolia toxicity’) to comprehensively retrieve relevant literature in Simplified Chinese, Traditional Chinese, and English. The crawled raw text data were then preprocessed and analysed using a large language model to identify and extract segments containing toxicity descriptions, with a focus on adverse effects, mechanistic pathways, and clinical manifestations. Specifically, the model was used to filter out irrelevant literature and extract candidate text spans, prioritizing sentences/paragraphs that explicitly reported toxic outcomes (e.g. organ injury, clinical symptoms, pathological findings) rather than generic statements. The model assisted in preliminary categorization and relevance scoring based on semantic similarity to toxicity-related concepts. Subsequently, all automatically processed records underwent rigorous manual curation. Curators confirmed entity identity (resolving synonyms/homonyms), verified that each included literature reported explicit toxicity under a clearly described exposure context, and excluded literature that merely mentioned ‘toxicity’ as a general property without evidence or where the studied material could not be confidently mapped to a specific formula or herb. Based on the Chinese Pharmacopoeia and international toxicology terminology standards, we performed multi-level validation: first screening titles and abstracts, then conducting full-text reviews to confirm explicit statements about toxicity. For quality control, a subset of curated records was cross-checked by an additional curator; disagreements were resolved by discussion and by prioritizing the most explicit evidence in the full text and authoritative terminology standards. Only literature with clear, evidence-based toxicity conclusions was incorporated into TCMToxDB.

### Search and browse

**Figure 2:**
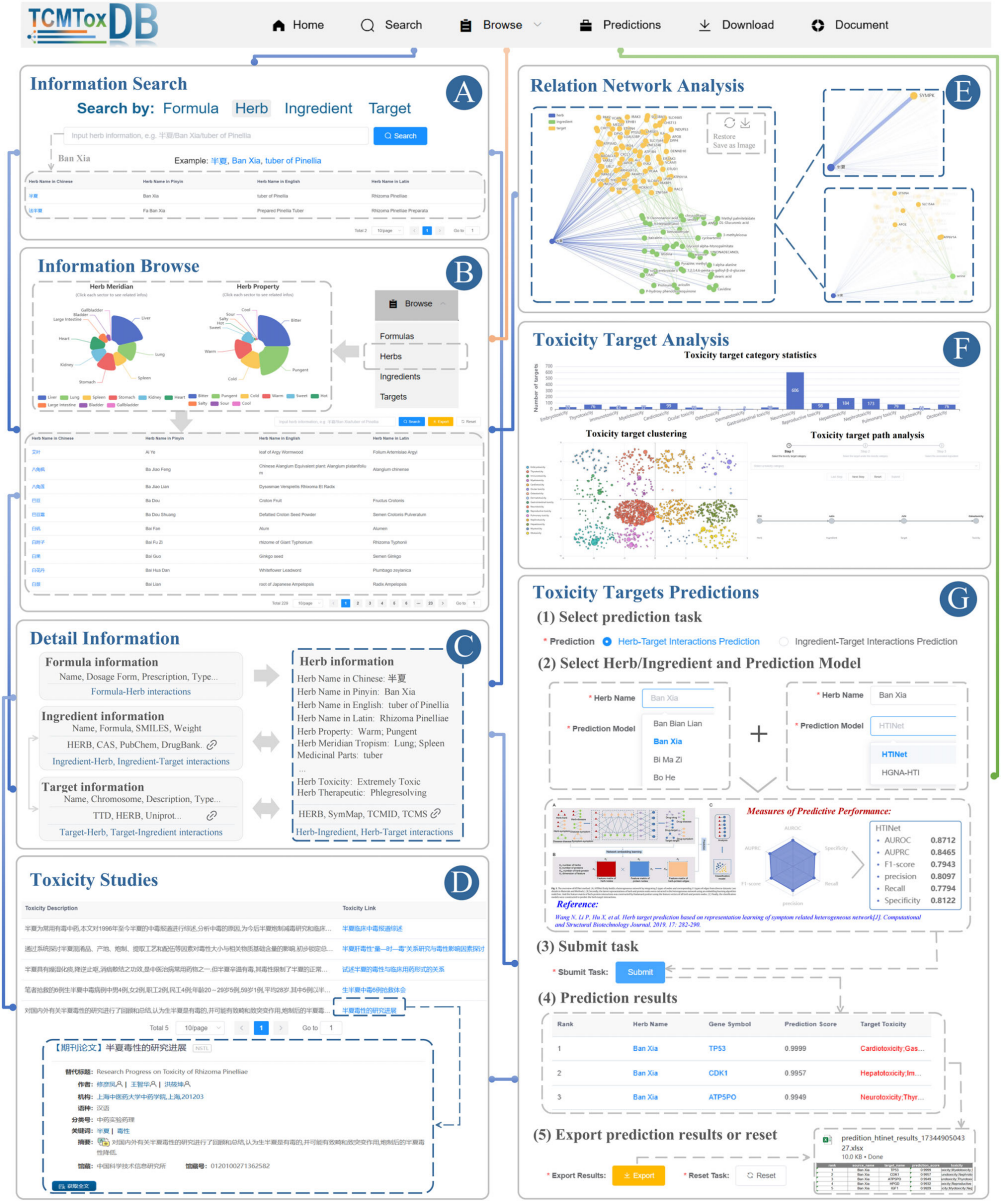
Usage of TCMToxDB. (A) Herbs can be searched via keywords such as Chinese Pinyin, Chinese characters, and Latin names. (B) Browse herb information, and click on pie fan leaf to view different types of data. (C) The summary panel on the detail page shows descriptive information for the formula, herb, ingredient, and target. (D) The related toxic results of TCMs. The corresponding literature description and links are given in the form of a list. (E) Network analysis of herbs, ingredients, and target interactions, with nodes coloured by their source component. Holding the mouse pointer over the node highlights the node and its related edges while showing its name. (F) Toxicity targets analysis. TCMToxDB provides three toxic target analysis functions, toxicity target category statistics, toxicity target clustering, and toxicity target path analysis. (G) Toxicity targets prediction. TCMToxDB provides two toxic target prediction tasks: (i) of herbs and (ii) of ingredients.

On the ‘Search’ page, users can search the four types of data provided by TCMToxDB, including formulas, herbs, ingredients, and targets. Users need to first select the types, and then fill in the relevant fields for the information they wish to search. The search box will give certain input reference examples; users can enter their own search information like the reference keywords. At the same time, some reference examples are also given under the search box, and users can click the reference example to enter the detailed information page. As shown in Fig. 2A, if the user wants to search information about the herb ‘Ban Xia’, the user can enter the English name of the herb ‘tuber of Pinellia’ or the Chinese name ‘Ban Xia’ in the search box and click the search button to search. After that, a table appears at the bottom of the page to display the retrieved results with hyperlinks, we can click the link to enter the detailed information page.

On the ‘Browse’ page, users can explore formulas, herbs, ingredients, and targets by clicking on the respective categories. TCMToxDB stores a total of 1555 formulas (217 toxic formulas), 229 toxic herbs, 9377 ingredients, and 7885 targets (2047 toxic targets), all of which can be queried, browsed, and downloaded by users. Upon clicking, users are directed to the corresponding page. Taking the herb browse as an example (Fig. 2B), users can access the herb information page via search, browsing, or by clicking a hyperlink in the navigation bar. The detailed information on the herb page includes a statistical pie chart and a detailed table. The pie chart displays classification statistics for herb properties and meridian tropism. Users can hover the mouse cursor over the segments of the pie chart to view the proportion of each category. Upon clicking a segment, the table below is updated to reflect the corresponding information. For instance, when the user clicks on the ‘Warm’ category under herb properties, herbs with Warm properties, such as ‘Ban Xia’, will appear in the table below. Additionally, users can further refine their search by entering keywords into the input box in the upper right corner of the form, with the option to either export the results or reset the page.

### Detail information

For each entry in the categories of formulas, herbs, ingredients, and targets, users can query detailed information. For herbs, basic information includes the Chinese name, Chinese pinyin name, English name, Latin name, properties, meridian tropism, medicinal parts, efficacies, indications, toxicity, cross-links to external databases (e.g. HERB, SymMap, etc.), herb–ingredient association, and herb–target association. For the formula, the basic information includes the Chinese name, Chinese pinyin name, syndrome, dosage form, formula type, prescription, diseases treated, indications, efficacy, and formula-toxicity herb association. For the ingredient, the basic information includes the name, molecular SMILES, molecular formula, molecular weight, cross-links to external databases (e.g. PubChem for chemical properties, bioactivity, and 3D structures), ingredient–herb association, and ingredient–target association. For the target, the basic information includes the name, gene symbol, target type, chromosome, map location, sequence, cross-links to external databases (e.g. UniProt for functional annotations, TTD for disease associations), target–herb association, and target–ingredient association. Among them, the associations of formula–herb, herb–ingredient, herb–target, and ingredient–target are presented in the form of tables, which list all relevant association information.

As shown in Fig. 2D, we collected existing literature on the toxicity of formula and herb as support. The toxicity-related records described in the literature are presented in the form of a list and links to the toxicity literature are provided for users to view the original text. In addition, as shown in Fig. 2E, the network diagram visualization analysis of herb–ingredient–target association is also provided, in which users can more intuitively view the association. When the mouse moves over a node or edge in the network diagram, the highlighted node or edge is displayed, and its information is displayed. Users can zoom in and out of the map at will, reset the map, or export it as an image by clicking the button in the upper right corner.

As shown in Fig. 2F, TCMToxDB provides a comprehensive toxicity target analysis function, including toxicity target category statistics, toxicity target clustering, and toxicity target path analysis. These functionalities are designed to systematically analyse toxic targets and provide insights into their underlying mechanisms, thereby facilitating a deeper understanding of toxicity-related processes.

For toxicity target category statistics, TCMToxDB not only provides toxicity targets associated with a selected herb but also performs a comprehensive analysis of their toxicity categories. The platform calculates and summarizes the number of targets within each category, providing a clear overview of the toxicity target categories associated with the herb. The results are displayed as a bar chart, where the *x*-axis represents toxicity categories and the *y*-axis shows the number of toxicity targets within each category, offering an intuitive way for users to explore the data and gain insights to support further research and analysis.

For toxicity target clustering, TCMToxDB utilizes the *K*-means clustering [43], with the value of *K* set to the number of toxicity target categories, to conduct an in-depth analysis of toxicity target categories. By analysing the similarities and relationships among toxicity targets, the platform organizes them into distinct clusters. This approach not only highlights the category affiliations of toxicity targets, but also reveals potential functional relationships and biological significance within and across clusters. The results are displayed as an interactive clustering scatter plot, where the *x* and *y* axes represent dimensionally reduced coordinates for visualization purposes, providing a visually intuitive way for users to explore the distribution and connections of toxicity targets. This enables a deeper understanding of toxicity mechanisms and supports the prioritization of research targets.

For toxicity target path analysis, TCMToxDB employs a stepwise selection process that guides users to generate the path through three stages. At each stage, the system dynamically updates and filters options to ensure a logical progression and seamless data flow. The validation mechanism ensures that each step is completed before proceeding, while rollback and reset functionalities provide flexibility for refining inputs. After the user submits the task, TCMToxDB analyses the selected toxicity data to construct an interactive toxicity target path map, where the coordinates represent spatial positioning for network layout visualization, revealing the relationships and pathways among toxicity categories, targets, ingredients, and herbs. This map helps users to uncover potential toxicity mechanisms, and enables researchers to effectively analyse toxicity data and identify key targets.

### Toxicity target prediction

There is a vacancy of web service that can perform automatic toxicity targets prediction. To fill this gap, TCMToxDB builds a toxicity target prediction module that provides users with personalized toxicity target prediction service. As shown in Fig. 2G, it implements a five-step workflow: task selection, data input and model selection, task submission, result visualization and analysis, and result export.

For herb toxicity target prediction, two models are offered: HTINet [26] and HGNA-HTI [27]. For ingredient toxicity target prediction, three models are provided: DeepDTA [28], GraphDTA [29], and DrugBAN [30]. The methodologies underlying these models are elaborated in subsequent sections. To ensure that the toxic target prediction is specifically related to toxicity rather than general pharmacological effects, TCMToxDB leverages a curated toxicity target dataset. The prediction models are trained exclusively on this toxicity-specific dataset, which contains experimentally validated herb–target and ingredient–target interactions where the targets are confirmed to be associated with toxic effects. This targeted training approach enables the models to distinguish between toxicity-related targets and those responsible for beneficial pharmacological activities. Once a model is selected, detailed information about the model, including its architecture, a brief introduction, performance metrics, and reference links, is displayed at the bottom of the interface.

In the task submission phase, users finalize their prediction by clicking the ‘Submit Task’ button. The results are displayed at the bottom of the interface as a ranked list of toxicity targets with prediction scores. For each predicted target, hyperlinks are provided, enabling users to access detailed information. The detailed information page includes cross-references to authoritative external databases such as UniProt [40] and the TTD [38], which allow users to directly access comprehensive functional annotations and therapeutic context. These predicted results can be further used for toxicity target path analysis. Based on the predicted data, TCMToxDB constructed an interactive toxicity target path map through a three-stage step-by-step selection process to visually present the relationships and pathways between toxicity categories, targets, ingredients, and herbs, helping users to deeply explore potential toxicity mechanisms and efficiently identify key targets. Users can also export the prediction results in Excel sheets for further analysis. Additionally, a reset option is available to allow users to configure and submit new tasks as needed.

### Data download

The download function of TCMToxDB enhances data accessibility. This feature allows users to download comprehensive information on formulas, herbs, ingredients, and targets for data mining. All basic information can be bulk downloaded from the ‘Download’ page. Additionally, users can select data of interest on the ‘Browse’ page and download it accordingly. The prediction results of toxicity targets and other analytical results can also be exported. These files are formatted as CSV files, Excel sheets, or images.

### Development tools and web interface

To ensure stable system performance, TCMToxDB is deployed on a high-performance Ubuntu server with Intel Xeon Gold 5118 CPU, NVIDIA RTX3090 GPU clusters, 512GB RAM, and high-storage-capacity hard drives. The front-end of TCMToxDB is developed using the Vue.js framework (https://vuejs.org) and Nginx web server (https://nginx.org), and the back-end is developed using the Spring Boot framework (https://spring.io/projects/spring-boot). The large-scale data of TCMToxDB are stored in a MySQL database (https://www.mysql.com). TCMToxDB has a user-friendly interface, and it is compatible with the most often-used browsers on computers or mobile devices.

### Case study demonstration

Notably, to demonstrate the utility and workflow of the TCMToxDB platform, we present a case study investigating a well-known Chinese medicinal herb, Ban Xia (Pinellia ternata, Rhizoma Pinelliae). The detailed content is reported in the *Case Study: Step-by-Step Toxicological Investigation Using TCMToxDB* section in the Supplementary Materials.

## Results

### Comparison with relevant services

The differences between TCMToxDB and other TCM-related databases in terms of data and functionality are summarized in Table 2. To provide a more explicit and quantitative comparison, we further include Supplementary Table S1 (scale statistics and reported relation types) and Supplementary Table S2 (average association density derived from Table S1). Explanatory notes for both tables are provided in the *Database scope and interpretation of Table S1 and Table S2* section of the Supplementary Materials. Compared to existing databases, at the technical architecture level, while these database platforms employ different implementation approaches, most adhere to standard web application frameworks. In terms of content, TCMToxDB primarily emphasizes toxicity data analysis specific to TCMs. It offers the comprehensive and general platform for analysing the toxicity data of TCM. By integrating existing research on the toxicity of formulas and herbs, TCMToxDB provides more reliable and comprehensive literature support for toxicity studies of these entities. Additionally, it has developed a toxicity target prediction tool, expanding its utility across a broader range of applications.

**Table 2:** Main characteristics of toxicity databases related to TCMs.

Database	Main characteristics	Relation with toxicity study of TCMs
CTD [16]	Links toxicological information across chemicals, genes, phenotypes, diseases, and exposures	Primarily focuses on chemical drugs, only a small amount of ingredient information can be queried
LiverTox [9]	Provides information on liver damage caused by drugs, dietary supplements, and herbs, and is closely linked to clinical research	Focuses mainly on liver injury related to herbs, with relatively limited data available
Hepatox [10]	Includes drugs, herbs, and clinical cases related to drug-induced liver injury	Focuses mainly on liver injury related to herbs, with relatively limited data available, and clinical data in the database is not accessible to the public
SIDER [17]	Includes adverse drug reaction frequency, classification, drug–target relationships, and mechanisms of adverse reactions	Primarily focuses on adverse chemical drug reactions
HIM [20]	Provides an overview of *in vivo* metabolism-related toxicity, ADME, and clinical studies of active ingredients in herbs	Focuses on metabolism-related toxicity *in vivo*
TCMID [13]	A comprehensive TCM database integrating drugs, diseases, targets, and prescriptions	Primarily focuses on pharmacology, with limited coverage of toxicity in TCM, providing toxicity labels for only certain herbs
TCM-ID [32]	Integrates herbs science with modern bioinformatics to promote herb certification and connect with healthcare big data	Primarily focuses on pharmacology, with limited coverage of toxicity in TCM, providing toxicity labels for only certain herbs
TCMSP [15]	Provides drug targets and associated diseases for each active ingredient, enabling the creation of ingredient–target and target–disease networks	Primarily focuses on pharmacology, with limited coverage of research on toxicity in TCM, providing toxicity labels for only certain herbs
SymMap [33]	Maps internal molecular mechanisms and external symptoms, combining herbs with modern medicine for drug screening	Primarily focuses on the mapping between TCM symptoms and modern symptoms, with limited coverage of TCM toxicity, providing toxicity labels for only certain herbs
TCMBank [19]	Allows users to explore the relationships between herbs, ingredients, gene targets, and related pathways or diseases, and predict adverse reactions of Chinese and Western medicines	Provides prediction algorithms for adverse reactions of TCMs and chemical drugs, without toxicity studies
ETCM [18]	A comprehensive resource for herbs or formulas that facilitates research of functions and mechanisms of TCMs	Focuses mainly on pharmacology, providing toxicity labels for only certain herbs
HERB [14]	Integrates pharmacogenomics data, links target–disease-ingredient relationships, supports the modernization of herbs, and guides drug discovery	Primarily integrates transcriptomic data, providing toxicity labels for only certain herbs
HIT [34]	A comprehensive search platform for ingredients and targets based on literature evidence	Focuses on herbal ingredient–target relationships
TCMToxDB	Systematically and comprehensively integrates and analyses the research results of toxic TCMs, provides the services of toxic target prediction of herbs and ingredients to empower TCM toxicity research	Integrates existing database records and manually curated literature data, along with five canonical algorithms to predict toxicity targets of herbs and ingredients, provides comprehensive and reliable toxicity information for TCMs

### Experimental setup

We extracted data from TCMToxDB on toxic herbs, targets, and ingredients. Specifically, we obtained data on 229 toxic herbs, 1033 ingredients, and 2047 toxic targets, as well as 22 073 toxic herb–target interactions and 3777 toxic ingredient–target interactions. Table 3 lists the details of these data sources, where efficacy, property, and meridian are attributes of herbs, and their quantities are 604, 12, and 30, respectively.

**Table 3:** Training data of toxicity target prediction methods.

Type	Size	HTINet [26]	HGNA-HTI [27]	DrugBAN [30]	DeepDTA [28]	GraphDTA [29]
Herb efficacy	229 × 604	✓	✓	–	–	–
Herb meridian	229 × 12	✓	✓	–	–	–
Herb property	229 × 30	✓	✓	–	–	–
Ingredient SMILES	1033	–	–	✓	✓	✓
Target sequence	2047	✓	✓	✓	✓	✓
Herb–target	22073	✓	✓	–	–	–
Ingredient–target	3777	–	–	✓	✓	✓

*Note:* The size of herb efficacy is 229 × 604, indicating that the number of herbs is 229 and the number of efficacy is 604. Herb meridian and herb property are represented in the same way as herb efficacy. ‘✓’ and ‘–’ indicate whether the data are used.

We use known toxic herb–target pairs and toxic ingredient–target pairs as positive samples, while unknown pairs as negative ones. The positive samples are collected from existing toxicological databases and experimental findings, ensuring they are reliable representations of true toxic interactions. The negative samples, on the other hand, represent pairs that are assumed to be non-toxic, though they may also include pairs that are uncharacterized. To maintain a balanced dataset, we perform negative sampling to ensure an equal number of positive and negative samples. Specifically, 15% of the data are reserved as the testing set, while the remaining data are randomly partitioned into five subsets, with four subsets used as training sets, and the other one as the validation set.

To evaluate the performance of these methods, we employ a suite of evaluation metrics, including AUROC (Area Under the Receiver Operating-Characteristic Curve), AUPRC (Area Under the Precision–Recall Curve), F1-score, precision, recall, and specificity. The ranges of them are all within [0, 1], and a higher value indicates a better performance.

### Toxicity target prediction methods

Recent advances in artificial intelligence have demonstrated the utility of machine learning in biomedical prediction [44–48]. Accordingly, TCMToxDB employs artificial intelligence algorithms to predict toxicity targets of herbs and ingredients, thereby elucidating the potential toxicological mechanisms of these substances. Specifically, we selected five prediction methods of herb–target and ingredient–target interactions for the prediction of toxic targets, including HTINet [26], HGNA-HTI [27], DeepDTA [28], GraphDTA [29], and DrugBAN [30]. The first two aim to infer herb–target interactions, while the latter three predict ingredient–target interactions. For these methods, we adopt their recommended parameter configurations or employ shared codes from their respective papers, fine-tuning them to align with our prediction tasks.

Among them, HTINet [26] models the heterogeneous associations between herbs, symptoms, drugs, diseases, and targets, then utilizes node2vec [49] and K-nearest neighbour [50] to predict targets. HGNA-HTI [27] employs meta-relationships and attention mechanisms to learn the topological structure and semantic information of herb–target heterogeneous graph, and to predict targets. DeepDTA [28] uses Convolutional Neural Networks to learn representations from the raw sequence data of targets and ingredients and fully connected layers in the affinity prediction task. GraphDTA [29] is capable of directly modelling ingredients as molecular graphs, then uses fully connected layers to predict targets of ingredients. DrugBAN [30] is a deep learning framework with explicit learning of local interactions between ingredients and targets, and conditional domain adaptation for learning transferable representations across domains.

The Supplementary Materials (section *Selected prediction models and their adoption in TCMToxDB*) further elaborate on the rationale for selecting these five representative methods and describe the task-specific adaptations in TCMToxDB.

### Herb toxicity targets prediction

For the prediction of herb toxicity targets, TCMToxDB integrates two algorithms, HTINet [26] and HGNA-HTI [27], and their performance on our constructed toxicity herb–target dataset is shown in Table 4. We can observe that both algorithms show strong prediction performance. Among them, HGNA-HTI is slightly stronger than HTINet, because the feature extraction based on random walk in HTINet cannot effectively mine the herb–target heterogeneous networks, and HGNA-HTI can aggregate the information of high-order neighbours more effectively by stacking multiple embedded propagation layers, using attention and message passing mechanism.

**Table 4:** Performance of toxic target prediction methods.

Prediction task	Model	AUROC	AUPRC	F1-score	Precision	Recall	Specificity
herb toxic	HTINet [26]	0.8712 ± 0.0042	0.8465 ± 0.0044	0.7943 ± 0.0054	0.8097 ± 0.0072	0.7794 ± 0.0084	0.8122 ± 0.0061
targets	HGNA-HTI [27]	0.9111 ± 0.0032	0.9108 ± 0.0014	0.8385 ± 0.0020	0.8134 ± 0.0048	0.8651 ± 0.0044	0.8225 ± 0.0044
ingredient	DrugBAN [30]	0.9611 ± 0.0046	0.9490 ± 0.0038	0.9039 ± 0.0051	0.9291 ± 0.0058	0.8800 ± 0.0056	0.9237 ± 0.0036
toxic targets	DeepDTA [28]	0.8486 ± 0.0051	0.6258 ± 0.0024	0.5662 ± 0.0033	0.6661 ± 0.0049	0.4923 ± 0.0062	0.6432 ± 0.0078
	GraphDTA [29]	0.9482 ± 0.0045	0.7810 ± 0.0035	0.7897 ± 0.0058	0.8070 ± 0.0064	0.7000 ± 0.0062	0.7936 ± 0.0061

**Figure 3:**
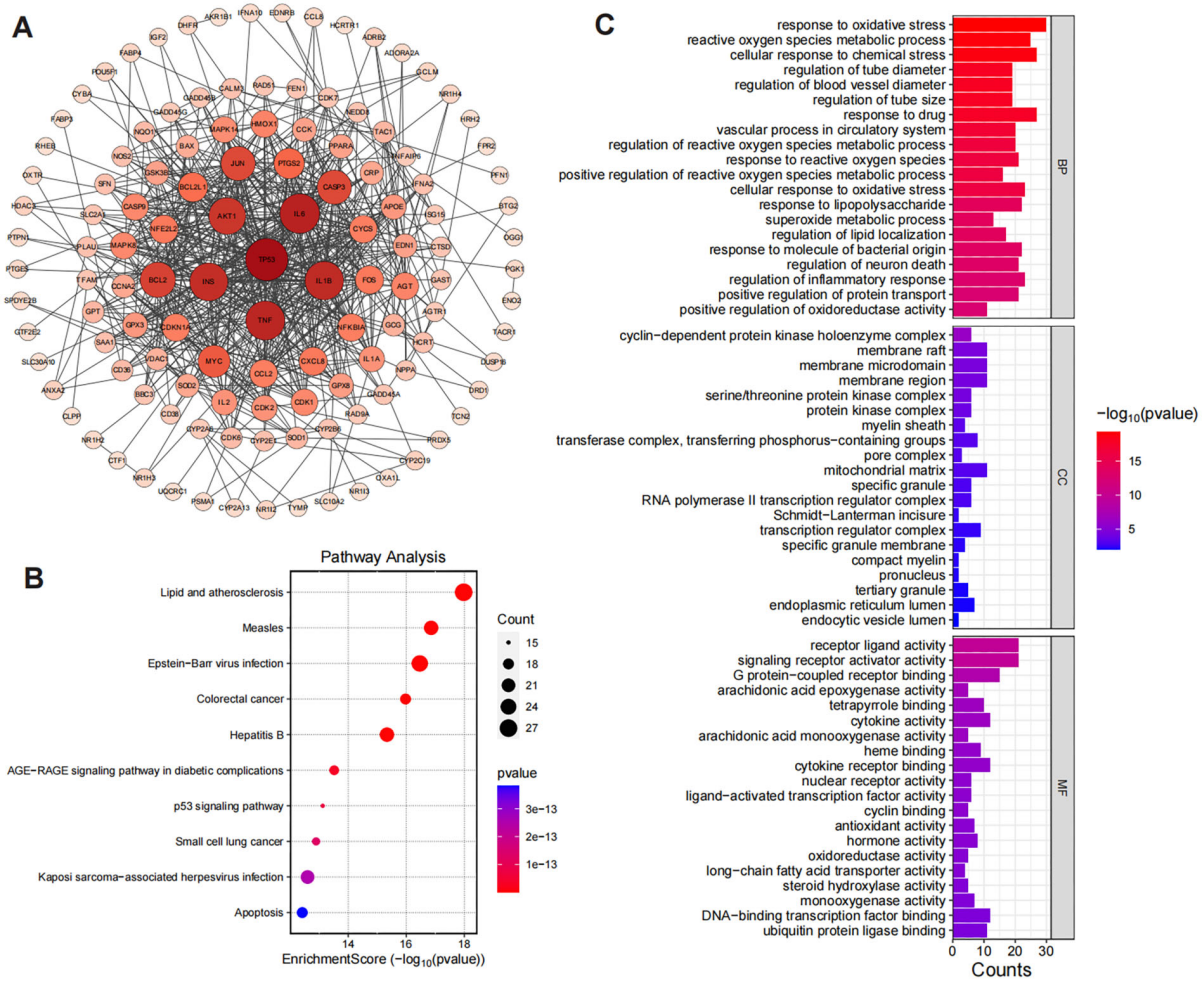
(A) The target–target interaction network of toxic targets predicted by HGNA-HTI. Node size and colour correspond to their respective degree values, with larger and deeper nodes representing higher degrees. (B) and (C) illustrate the top 20 enriched KEGG pathways and GO entries with smaller FDR values, respectively. The smaller the FDR value is, the higher the significance is. The size of each bubble corresponds to gene expressions in a particular pathway, and the colour saturation of the bubble reflects the enrichment significance.

To further analyse the prediction results, we take the herb *Xi Xin* as an example. Specifically, we used HGNA-HTI to predict the toxicity targets of *Xi Xin*, and a total of 143 toxicity targets were predicted. Next, we constructed a toxicity target–target interactions network based on the interrelationships between these toxicity targets, which consisted of 143 nodes (toxicity targets) and 599 edges (target–target interactions), with an average node degree of 8.15, as shown in Fig. 3A. Among the predicted toxicity targets, *TP53, IL6*, and *TNF* exhibit the highest degree values. Specifically, *TP53* is associated with a wide range of toxicities, including cardiotoxicity, gastrointestinal toxicity, hepatotoxicity, immunotoxicity, myelotoxicity, nephrotoxicity, neurotoxicity, ocular toxicity, and pulmonary toxicity. *IL6* is primarily linked to pulmonary toxicity and thyrotoxicity, while *TNF* is similarly associated with pulmonary toxicity and thyrotoxicity.

To investigate the biological function of toxicity targets, we conducted Gene Ontology (GO) enrichment analysis and Kyoto Encyclopedia of Genes and Genomes (KEGG) pathway enrichment analysis, respectively. GO enrichment analysis covered biological processes (BPs), cellular components (CCs), and molecular functions (MFs) to elucidate the main biological functions. Additionally, KEGG enrichment analysis identified significant pathways associated with toxicity targets to unveil the primary pathways.

Fig. 3B illustrates the enriched entries for the KEGG pathways with smaller FDR values. KEGG enrichment analysis reveals that the targets are significantly enriched in multiple pathways related to inflammation, apoptosis, and metabolic dysregulation, including lipid and atherosclerosis, p53 signalling pathway, AGE-RAGE signalling pathway in diabetic complications, and several pathways associated with viral infections (e.g. measles, Epstein–Barr virus infection, and hepatitis B). The involvement of key targets in the lipid and atherosclerosis pathway, such as *IL6, TNF, CXCL8*, and *TP53*, suggests that these targets may contribute to the progression of inflammatory diseases by mediating cytokine signalling and vascular damage [51]. Similarly, the enrichment of targets in the p53 signalling pathway (e.g. *TP53, CASP9*, and *BAX*) highlights their pivotal roles in DNA damage-induced cell cycle arrest and apoptosis [52]. Moreover, the enrichment in the AGE-RAGE signalling pathway in diabetic complications, with inflammatory mediators like *IL6, TNF*, and *CCL2*, indicates the potential involvement of these targets in regulating inflammation and oxidative stress caused by metabolic disorders [53]. Notably, the enrichment in viral infection-related pathways (e.g. Epstein–Barr virus infection and hepatitis B) further suggests that these targets may influence disease progression through modulation of immune responses and host–pathogen interactions [54]. These pathways collectively encompass critical BPs in inflammation, immunity, and metabolic regulation, while also revealing interconnections between pathways, such as the crosstalk between the p53 signalling pathway and the apoptosis pathway via key targets like *TP53* and *CASP9*, which jointly regulate apoptotic processes [55]. This study provides valuable insights into the potential mechanisms of targets in various diseases and lays a foundation for further investigation into their specific functional roles within these pathways.

The results of GO enrichment analysis (Fig. 3C) reveal significant associations with several key biological categories, providing deeper insights into the potential mechanisms underlying the observed biological effects. (i) BPs: The analysis highlighted several enriched BPs related to oxidative stress and reactive oxygen species regulation. Notably, terms such as response to oxidative stress, reactive oxygen species metabolic process, and cellular response to chemical stress were highly significant. These processes are critical in maintaining cellular homeostasis under stress conditions, suggesting that the identified targets might play key roles in cellular protection against oxidative damage. Targets such as *DHFR, CYCS, TP53*, and *SOD1* were frequently involved, indicating their central role in stress response mechanisms and their potential therapeutic relevance in diseases associated with oxidative damage [56]. (ii) CCs: In the context of cellular localization, several enriched terms were identified, including the membrane raft, membrane microdomain, and mitochondrial matrix. These components are essential for signal transduction and cellular communication, with the mitochondrial matrix being particularly significant for cellular energy metabolism and stress response. Targets such as *VDAC1, SOD1*, and *TP53* were implicated in these processes, highlighting their potential involvement in mitochondrial function and oxidative stress regulation [57]. (iii) MFs: The MF enrichment revealed significant terms such as receptor–ligand activity and G protein-coupled receptor binding. These functions are crucial for intercellular communication and signal transduction. The involvement of targets like *IL6, CCL2*, and *CXCL8* suggests a strong link to immune response pathways and inflammation, further emphasizing the role of the identified targets in regulating inflammatory and immune-related responses [58]. Overall, these findings pave the way for further investigating how those targets contribute to disease progression and therapeutic interventions.

### Ingredient toxicity targets prediction

Taking the toxicity ingredient *methyl eugenol* of *Xi Xin* as an example, we used the DrugBAN algorithm integrated in TCMToxDB to predict its potential toxic targets. *Methyl eugenol* is a natural ingredient categorized as a phenylpropene, a subclass of phenylpropanoids. Existing researches indicate that *methyl eugenol* can induce cytotoxicity and genotoxicity in rodents, possibly due to sulfotransferase-mediated reactive metabolite formation, leading to liver injury [59].

**Figure 4:**
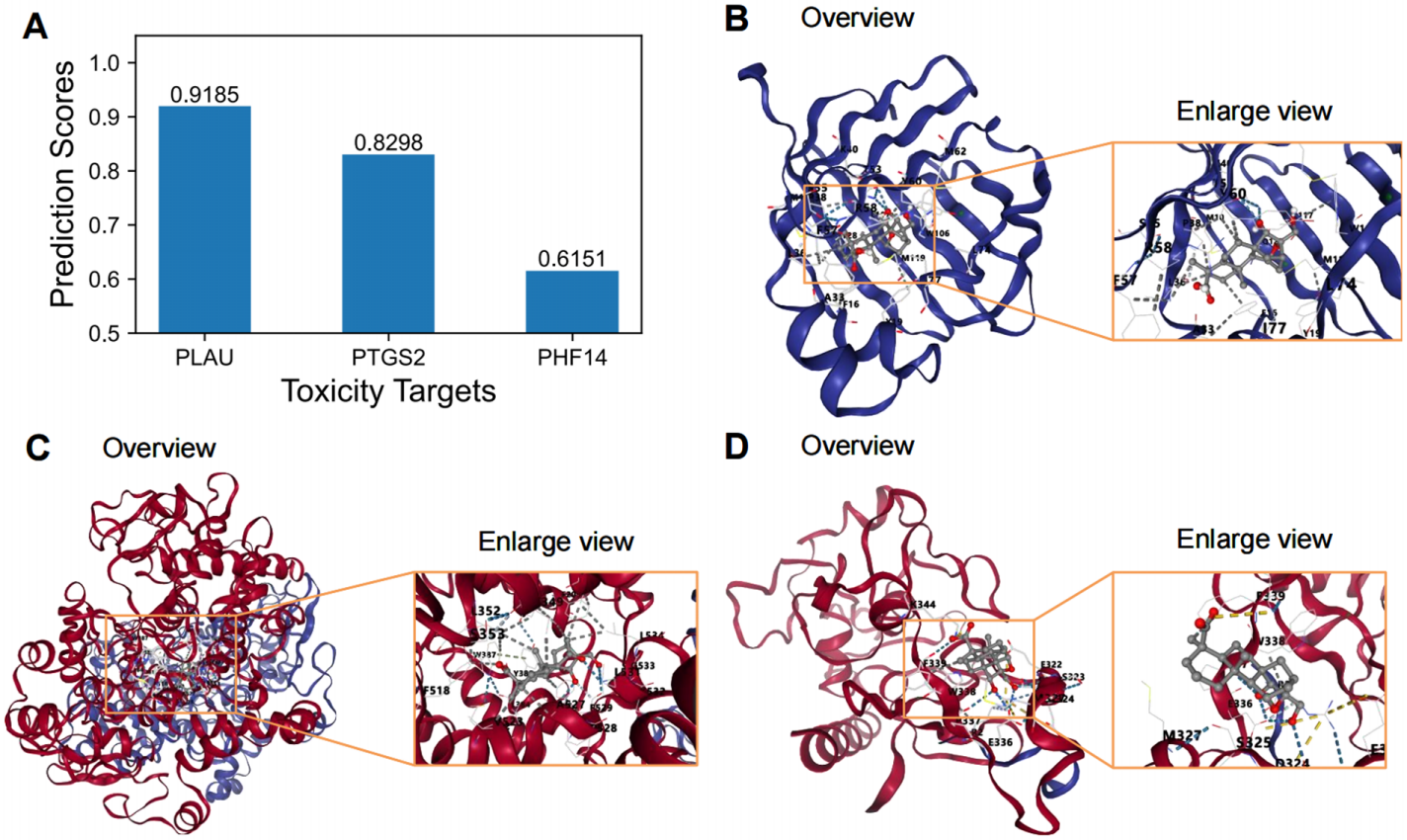
Molecular docking results with the lowest binding energy between ingredient methyl eugenol and toxicity targets predicted by DrugBAN. (A) Predicted toxic targets (PLAU, PTGS2, and PHF14) of ingredient methyl eugenol by DrugBAN, (B) eugenol-PLAU, (C) methyl eugenol-PTGS2, and (D) methyl eugenol-PHF14.

The top three predicted toxic targets of *methyl eugenol* were identified as *PLAU, PTGS2*, and *PHF14*, respectively (Fig. 4A). (i) *PLAU* (plasminogen activator, urokinase) is primarily responsible for the activation of plasminogen, facilitating extracellular matrix degradation. This process is biologically implicated in thrombosis, inflammation, cancer invasion, and metastasis. Clinically, *PLAU* is targeted in anticancer therapies and thrombolytic treatments [60]. (ii) *PTGS2* (prostaglandin-endoperoxide synthase 2, also known as COX-2) is a critical enzyme in the biosynthesis of prostaglandins and plays a key role in the regulation of inflammatory responses. Biologically, it is involved in inflammatory diseases, cancer progression, and cardiovascular disorders. Clinically, *PTGS2* is targeted by nonsteroidal anti-inflammatory drugs and anticancer treatments [61]. (iii) *PHF14* (PHD finger protein 14) is a chromatin-associated protein involved in epigenetic regulation and chromatin remodelling. It is biologically associated with fibrotic diseases, cancer, and developmental anomalies. *PHF14* serves as a potential target for fibrosis therapy and epigenetic interventions [62]. These findings highlight the relevance of *methyl eugenol*’s toxic targets in diverse BPs and clinical applications, providing valuable insights into its toxicological profile.

Finally, we conducted a molecular docking experiment to further study the association between the predicted toxic targets and ingredient *methyl eugenol*. Table 5 and Fig. 4 show the experimental results of the molecular docking, which demonstrate that *methyl eugenol* exhibits varying binding affinities with three toxicity targets: *PLAU, PTGS2*, and *PHF14*. Among them, *PTGS2* shows the highest binding affinity with a binding energy of −9.2 kcal/mol, followed by *PLAU* at −8.7 kcal/mol, and *PHF14* at −6.8 kcal/mol. The docking results show that *PTGS2* possesses a significantly larger binding cavity (4431 A³) compared to *PLAU* (1061 A³) and *PHF14* (733 A³), suggesting that *PTGS2* offers a more flexible and accommodating binding site for *methyl eugenol*. Notably, the *PTGS2* binding site, centred at coordinates (14, 49, 65), provides a larger docking space (26 × 29 × 29 A³) than the other two targets, which may contribute to the stronger binding interactions. In contrast, the compact binding sites of *PLAU* and *PHF14* indicate a more rigid docking environment, potentially limiting their binding interactions with *methyl eugenol*.

**Table 5:** Molecular docking results of ingredient *methyl eugenol* with toxic targets predicted by DrugBAN [30].

Target name	Rank	Binding energy (kcal/mol)	Cavity volume (A³)	Left (*x, y, z*)	Docking size (*x, y, z*)
PLAU (plasminogen activator, urokinase)	1	−8.7	1061	(−15, −3, 16)	(20, 20, 20)
	2	−6.6	93	(−25, −2, 17)	(20, 20, 20)
	3	−5.7	92	(−18, −15, 9)	(20, 20, 20)
	4	−5.7	90	(−12, −2, 26)	(20, 20, 20)
	5	−5.3	62	(−18, −16, 2)	(20, 20, 20)
PTGS2 (prostaglandin-endoperoxide synthase 2)	1	−9.2	4431	(14, 49, 65)	(26, 29, 29)
	2	−8.9	1167	(31, 42, 38)	(20, 20, 20)
	3	−8.7	26146	(22, 39, 36)	(35, 35, 35)
	4	−6.6	1234	(32, 37, 18)	(20, 20, 32)
	5	−6.1	1233	(28, 28, 59)	(20, 27, 33)
PHF14 (PHD finger protein 14)	1	−6.8	733	(0, 4, 7)	(20, 20, 20)
	2	−6.7	1480	(7, 16, −11)	(20, 20, 20)
	3	−6.5	306	(8, 26, 4)	(20, 20, 20)
	4	−6.4	132	(−1, 24, −15)	(20, 20, 20)
	5	−5.8	619	(−1, 34, −4)	(20, 20, 20)

*Note:* For each pair, the molecular docking results with the top five binding energy are listed.

Biologically, the strong affinity of *methyl eugenol* for *PTGS2* suggests its potential to inhibit *PTGS2* activity, a key enzyme involved in inflammation and tumorigenesis, highlighting its possible anti-inflammatory and anticancer properties [63]. The interaction with *PLAU* may implicate *methyl eugenol* in modulating fibrinolysis or tumour invasion pathways, while its weaker interaction with *PHF14* could influence gene expression regulation with less pronounced effects. These findings highlight that the algorithms integrated in TCMToxDB can accurately predict the toxic targets of ingredients and herbs, and provide data support for biologists to perform subsequent toxic molecular mechanism analysis.

## Discussion

Based on the systematic interactions among herbs, ingredients, and targets, the ‘herb–ingredient–target’ network was constructed, providing a novel methodology for understanding the pharmacology and mechanisms of TCMs. However, despite the development of several TCM databases using network-based pharmacology in recent years [14, 19, 34], these databases often share similar functionalities but differ in data sources, lack standardization, and comprehensiveness, and fail to incorporate toxicity analysis of TCMs. Therefore, this study aims to establish a more robust and comprehensive toxicological analysis database for TCMs, named TCMToxDB, by integrating scattered resources and manually curated toxicity-related data from published literature. TCMToxDB is a free and open-source database designed to offer a comprehensive resource for analysing toxicity in TCM, covering formulas, herbs, ingredients, targets, and relevant toxicity literature. Additionally, it provides a toxicity target prediction service, enabling users to predict the toxic targets of different herbs or ingredients with interest. Thus, TCMToxDB aims to systematically integrate and analyse the research results of toxic TCMs, and to provide the services of toxicity analysis and target prediction of herbs and ingredients. TCMToxDB can boost research on toxicity in TCM. We also plan to integrate an LLM component as an optional explanation layer to improve interpretability of our results, thereby making our data more comprehensible to users. Despite this, TCMToxDB still faces some challenges, such as its primary focus on the analysis of toxic TCMs, with relatively limited research on non-toxic TCMs and chemical drugs. In future updates, we plan to address this by integrating additional systematic medical databases, such as MalaCards [64], with the TCM database, thereby bridging the gap between TCMs and chemical drug research. Meanwhile, some toxic herbs or ingredients in the database still lack any annotated molecular targets, which is a challenge faced across the entire field. We plan to narrow this gap using machine learning algorithms to infer putative toxic targets for these entities in the future. Moreover, data updates and algorithm upgrades are crucial tasks for TCMToxDB, ensuring that the platform reflects the latest advancements in research on toxicity in TCM and highlights its ongoing relevance. Consequently, TCMToxDB is updated annually, with new versions released on December 30th each year. Each release is assigned a unique version number to ensure reproducibility and proper citation by users. Version control allows researchers to access and cite specific database releases, ensuring reproducibility of their analyses. We continuously monitor developments in the research on toxic TCMs, promptly remove outdated data, and supplement missing information with the latest research findings to enhance the platform’s functionality and ensure it aligns with real-world needs. We pay a close attention to the platform’s status at all times to detect and address any issues, ensuring users can seamlessly utilize TCMToxDB. In future updates, we will continue to refine the algorithms and software based on advancements in computing and the evolving needs of researchers studying toxicity in TCM.

TCMToxDB represents an important advancement in the field of research on toxicity in TCM, offering a comprehensive and user-friendly platform for analysing and predicting the toxicological properties of herbs and their ingredients. TCMToxDB aims to bridge gaps in current TCM research and provide a more holistic understanding of TCM’s pharmacological mechanisms by integrating diverse data sources and providing advanced services, such as toxicity targets analysis and prediction. While challenges remain, particularly in expanding the scope of research to include non-toxic TCMs and chemical drugs, future updates will continue to refine and enhance the platform, ensuring it remains an essential tool for researchers and practitioners in the field of TCM toxicity.

## Conclusion

TCMToxDB is a comprehensive database focusing on the safety and toxicological analysis of TCM, providing innovative tools for analysing and predicting the toxicological properties of herbs and their ingredients. The database’s key innovations lie in two main aspects. First, it systematically integrates scattered resources, manually curated toxicity-related TCM data from published literature, and conducts comprehensive toxicity data analysis. Second, it combines five influential algorithms to predict toxic targets, offering insights into the potential toxicity effects of herbs and ingredients. By integrating diverse data sources and performing in-depth toxicity data analysis, TCMToxDB offers a comprehensive and user-friendly service that bridges the gap between traditional and modern approaches to understand TCM’s pharmacological mechanisms and toxicity. We are confident that TCMToxDB will be an effective tool for boosting the study of safety of TCMs and providing an efficient and comprehensive platform to professionals and practitioners in the fields of traditional medicine, pharmacology, toxicology, and biomedical research for sharing information on TCM toxicology.

## Supplementary Material

baag019_Supplemental_File
