# Supplementary material for: TCMToxDB: a comprehensive database for the toxicological analysis of traditional Chinese medicines
Source: Database (Oxford). 2026 Apr 16;2026:baag019. doi: 10.1093/database/baag019 (PMC13083690; doi:10.1093/database/baag019)
Supplement: baag019_Supplemental_File [file baag019_supplemental_file.pdf]

Database, 2026, pp. 1–13

doi: DOI HERE

Advance Access Publication Date: Day Month Year

Supplementary Information for TCMToxDB: A Comprehensive Database for the Toxicological Analysis of Traditional Chinese Medicines

Yongzheng Zhu,[1,2,†] Laihao Fang,[1,2,†] Yunbo Miao,[1,2] Longfei Ma,[1,2] Rong Sun,[3] Yimin Mao,[4] Wei Guo,[2] Jun Wang[2] and Guoxian Yu[1,2,*]

[1]School of Software, Shandong University, 1500 Shunhua Road, 250101, Shandong, China, [2]SDU-NTU Centre for Artificial Intelligence Research, Shandong University, 1500 Shunhua Road, 250101, Shandong, China, [3]The Second Hospital of Shandong University, Shandong University, 247 North Park Street, 250000, Shandong, China and [4]Renji Hospital, Shanghai Jiao Tong University, 160 Pujian Road, 200127, Shanghai, China

*Corresponding author. guoxian85@gmail.com

†These authors contributed equally to this work.

Case Study: A Step-by-Step Toxicological Investigation Using TCMToxDB

To demonstrate the utility and workflow of the TCMToxDB platform, we present a case study investigating a well-known Chinese medicinal herb, Ban Xia (ternata of Pinellia, Rhizoma Pinelliae). Ban Xia is extensively employed to treat coughs and nausea, yet is also noted for its potential toxicity, necessitating processing prior to clinical use. This renders it an ideal candidate for illustrating how researchers may utilise TCMToxDB to explore and predict potential toxicological mechanisms.

Information Retrieval and Initial Exploration

Direct Search

For a researcher who has a specific formula, herb, ingredient or target in mind, the most direct method is the "Search" page. As shown in Figure S1, the user can select the category of their query—in this case, "Herb". The system is flexible, accepting various identifiers as input. In this example, the user enters the pinyin name "ban xia" into the search bar, yielding two results: "tuber of Pinellia" and "Prepared Pinellia Tuber".

Browsing by Formula

Alternatively, a user might discover Ban Xia contextually. On the "Browse" page, users may access the formulae section. Here, they can filter for toxic or non-toxic formulae, browse or search for formulae treating specific conditions (Figure S2). By identifying formulae such as Er Chen Wan, users discover its efficacy for coughs and, upon examining its composition, recognize Ban Xian as a key constituent (Figure S3). This context-driven discovery prompts users to explore the herb's properties in greater depth.

Browsing and Searching for the Herb

Users may directly click the Ban Xia link within the formula, or access the "Browse Herbs" section and search for "Ban Xia" to view its details. Within this page, users may filter herbs by selecting a specific section of the pie chart representing the herb's meridian or property(Figure S4). The herb details page provides a comprehensive overview. On this interface, users can view the herb's fundamental information, literature related to its toxicity, ingredients, and targets (as shown in Figure S5) . Additionally, it displays relationship diagrams between the herb, its ingredients, and targets; statistics of toxicity target categories; clustering diagrams; and toxicity target pathway analyses (as shown in Figure S6). In particular, we can observe from Figure S5 that the indications for Pinellia ternata include: cough and asthma with abundant phlegm, vomiting nausea, glomus in chest, dizziness, headache, vexation and agitation in night, goiters with phlegm node, toxic swelling of sores and abscesses. It can also remove damp and phlegm, relieve nausea and vomiting, and eliminate stuffiness in the chest and the epigastrium. Crucially, a "Herb Toxicity" label clearly indicates its known toxic potential, directing the researcher's focus.

Exploring Ingredients and Known Targets

From the detailed page for Ban Xia, the researcher can seamlessly navigate to its constituent ingredients. TCMToxDB provides a list of known chemical compounds in Ban Xia, complete with structural information and links to other databases. Figure S7 shows the details of ingredient Istidina associated with Ban Xia. Furthermore, the user can explore the Targets already associated

© The Author 2026. Published by Oxford University Press. All rights reserved. For permissions, please e-mail: journals.permissions@oup.com

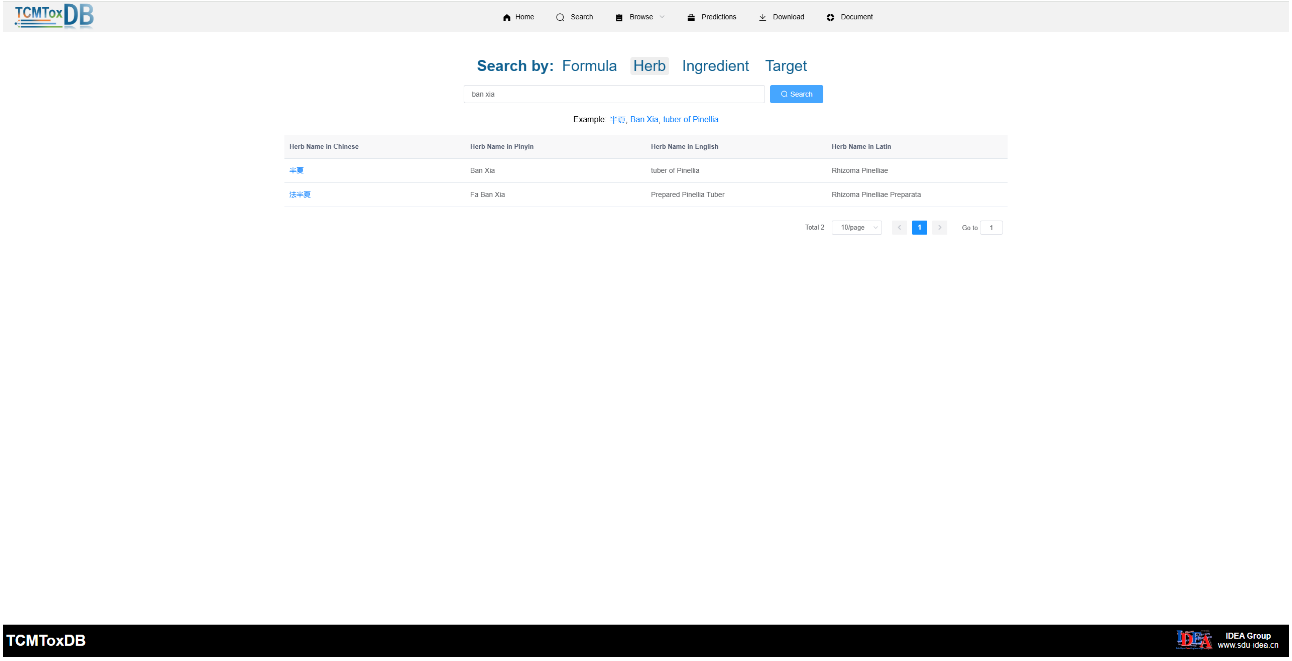

Figure S1. Page “Search”. The page displays search results for “ban xia”.

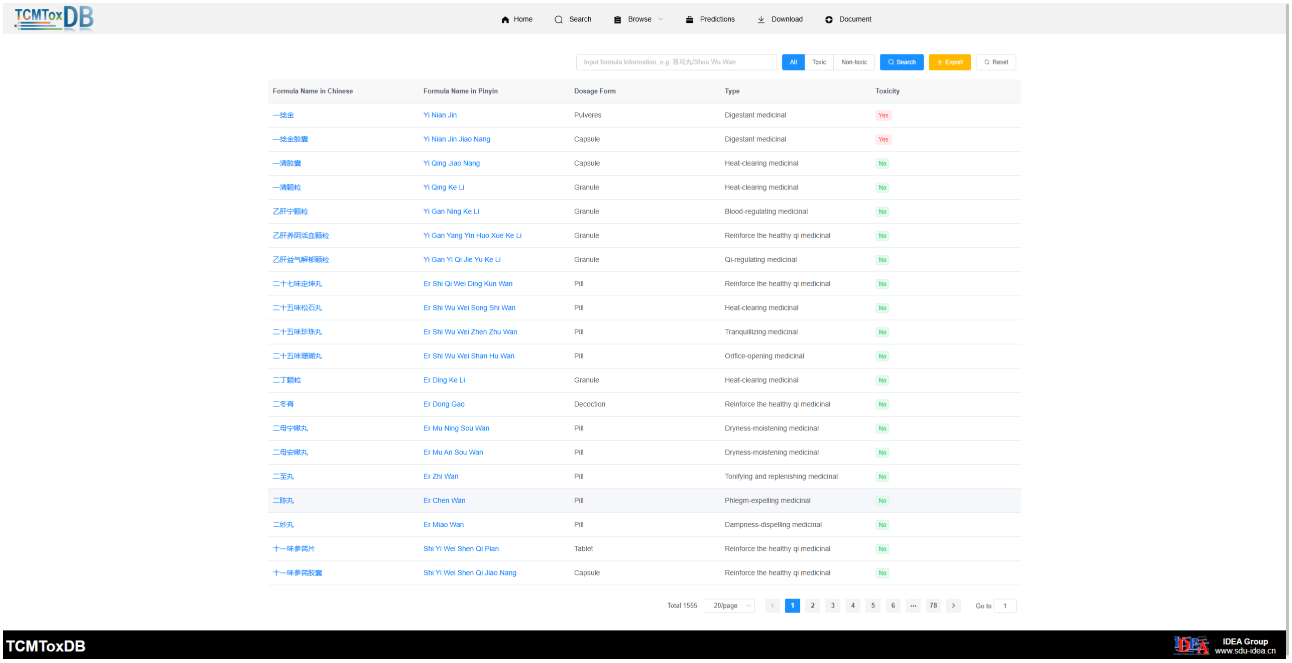

Figure S2. Page “Browse Formulas”. Enter the desired formula in the input field. The triple-filter box allows selection of all formulas, toxic formulas, or non-toxic formulas.

with Ban Xia in existing literature. This allows the user to review established knowledge before venturing into predictive analysis, forming a baseline for their investigation. Figure S8 shows the details of target CACNA1S associated with Ban Xia.

Predictive Analysis of Herb-Target and Ingredient-Target Interactions

To understand the potential mechanisms behind Ban Xia’s toxicity, the user proceeds to the “Predictions” module.

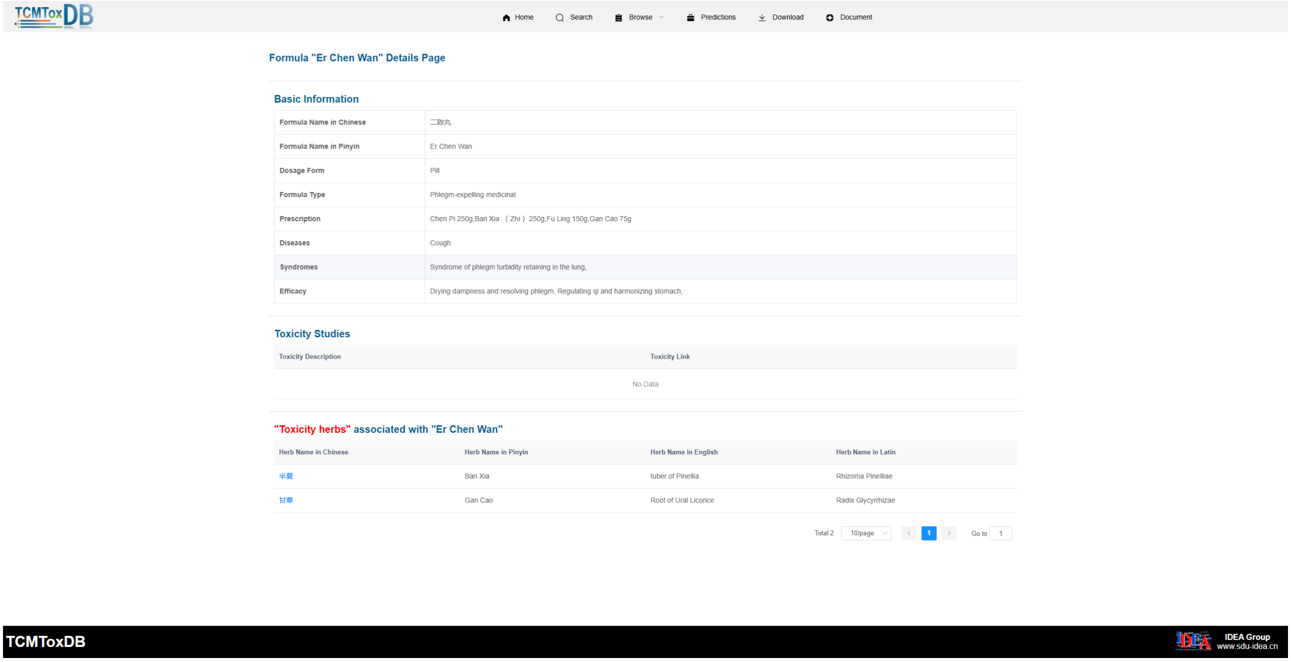

Figure S3. Page "Detail of Er Chen Wan". The page displays the basic information of the formula, toxicity studies related to the formula, and the toxicity herbs associated with the formula.

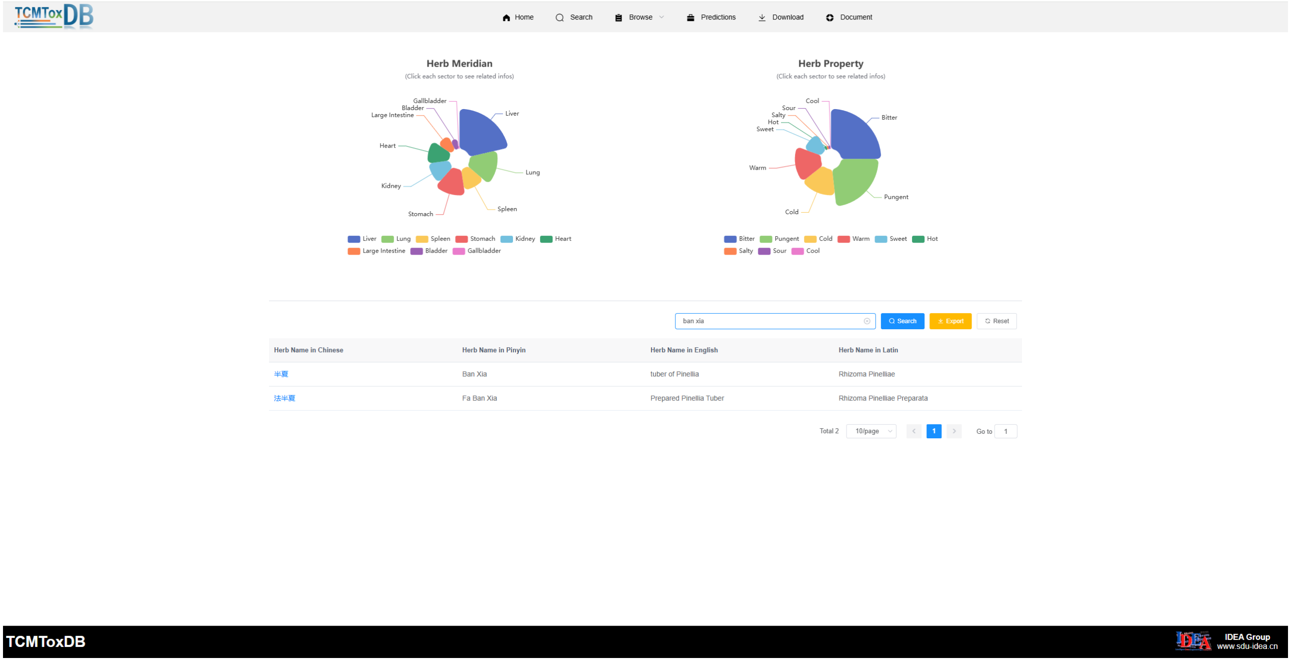

Figure S4. Page "Browse Herbs". Users may filter herbs by selecting a specific section of the pie chart representing the herb's meridian or property.

Task and Model Selection

The user selects the "Herb-Target Interactions Prediction" task. Enter "Ban" as a keyword to search for target herbs, then select "Ban Xia" from the dropdown suggestions for prediction (as shown in Figure S9). The platform offers several advanced prediction models, such as HTINet and HGNA-HTI. For this case study, we select HTINet. The interface conveniently displays the model's predictive performance metrics (e.g., AUROC: 0.8712±0.0042, AUPRC: 0.8465±0.0044), allowing the user to make an informed choice based on established benchmarks.

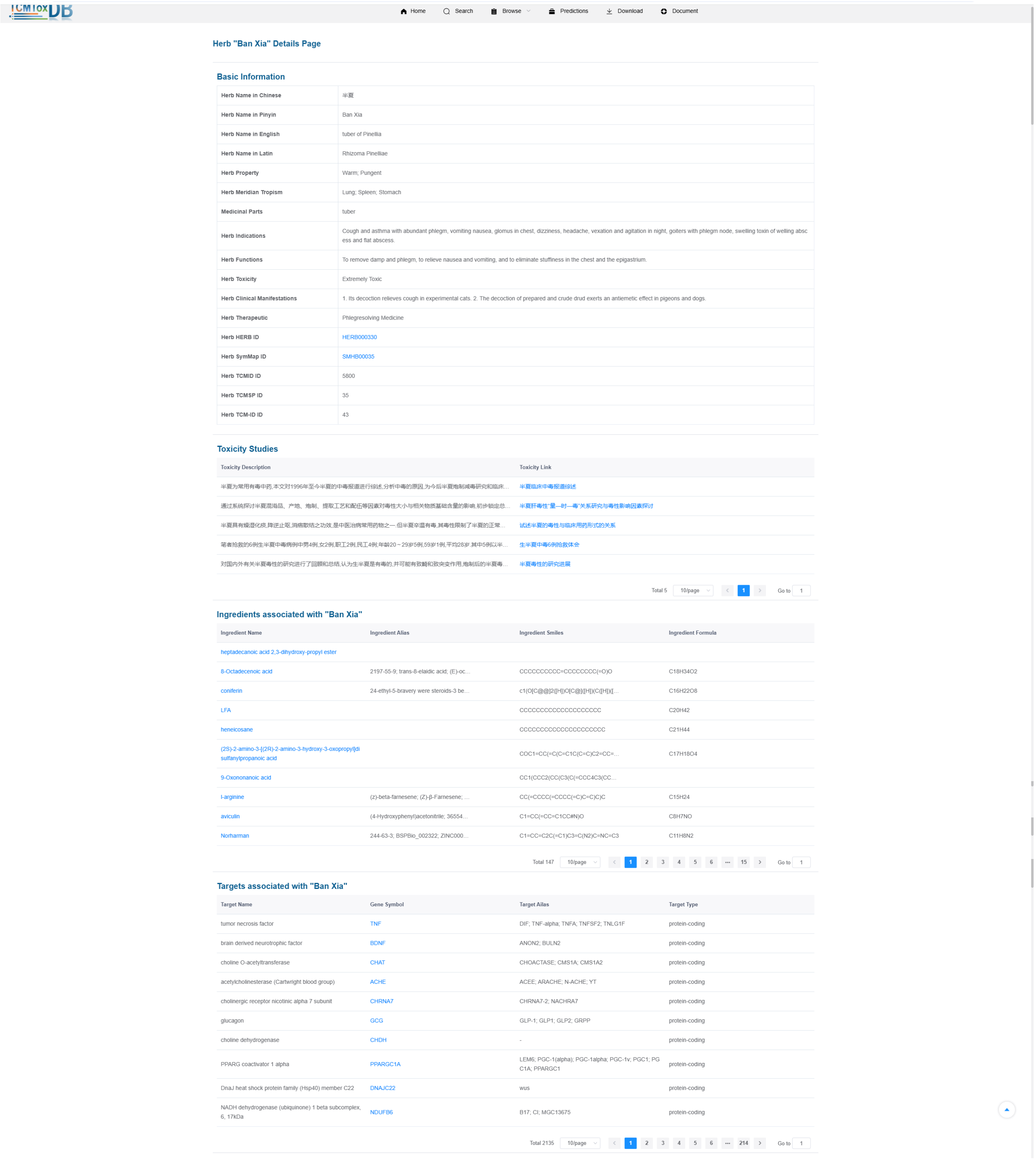

Figure S5. The upper section of the "Details of Ban Xia" page. This section displays the basic information of the herb Ban Xia, toxicity studies related to Ban Xia, and the toxicity ingredients and targets associated with Ban Xia.

Generating and Analyzing Prediction Results

After submitting the task, TCMToxDB rapidly generates a ranked list of putative toxic targets for Ban Xia. The results page (as shown in Figure S10) displays the predicted targets, their gene symbols, a prediction score indicating the confidence of the interaction, and the associated "Target Toxicity." In our analysis of Ban Xia, the top predicted targets include TP53 (Prediction Score: 0.9999), CDK1 (Prediction Score: 0.9957), ATP5PO (Prediction Score: 0.9949). These high-confidence predictions provide the researcher with specific, testable hypotheses.

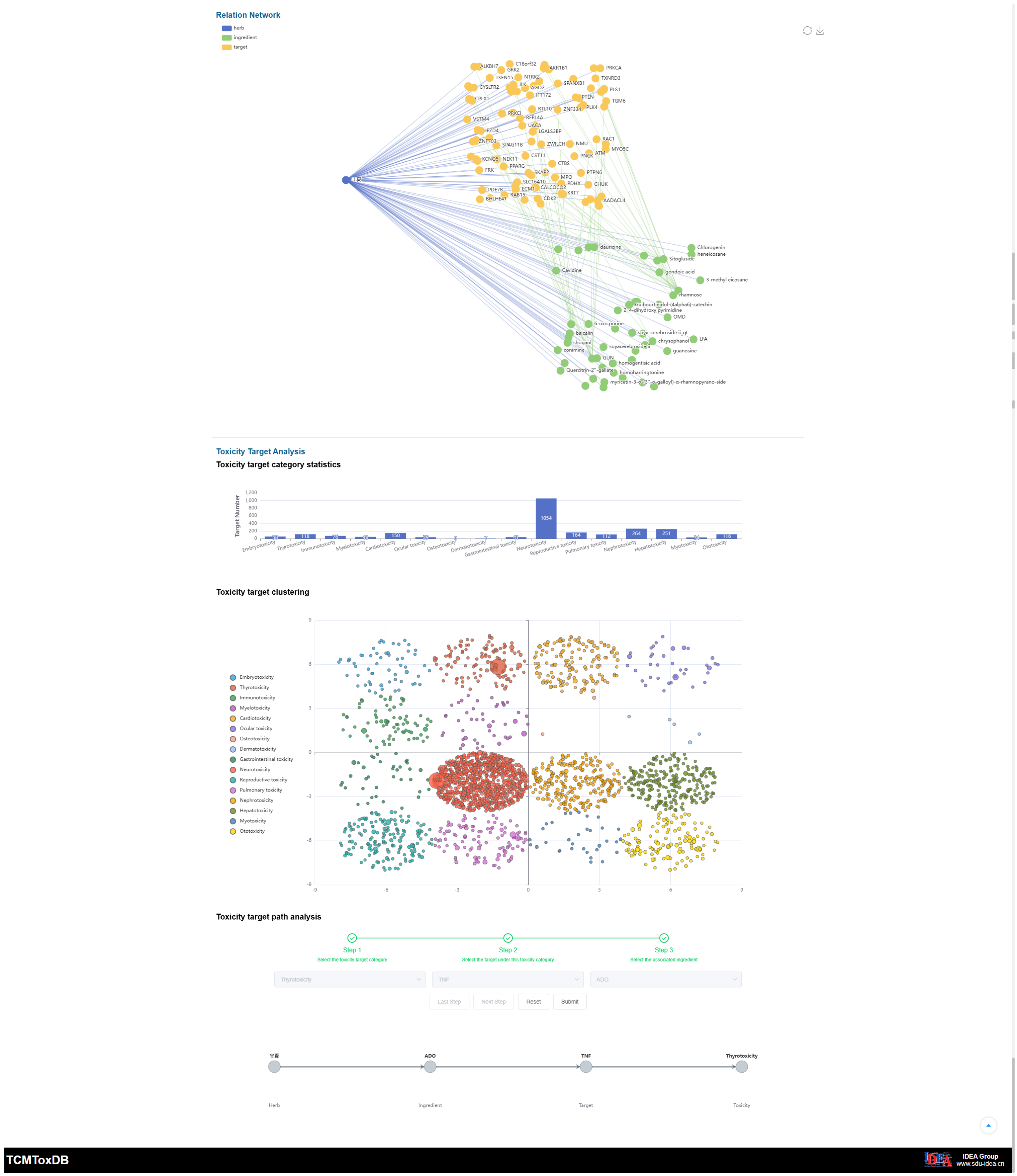

Figure S6. The lower section of the "Details of Ban Xia" page. This section displays the relation network of Ban Xia, statistical data on the number of targets associated with each category of toxicity related to Ban Xia, clustering of targets associated with each category of toxicity related to Ban Xia, and toxicity target pathway analyses.

Granular Analysis of Ingredient-Target Interactions

To further explore the underlying mechanisms, the researcher can investigate which specific ingredient(s) in Ban Xia might be responsible for these interactions. The user selects the "Ingredient-Target Interactions Prediction" task in the "Predictions" module. Based on the ingredient list for Ban Xia, the user chooses "Istidina" as the compound of interest, and DrugBAN as an appropriate

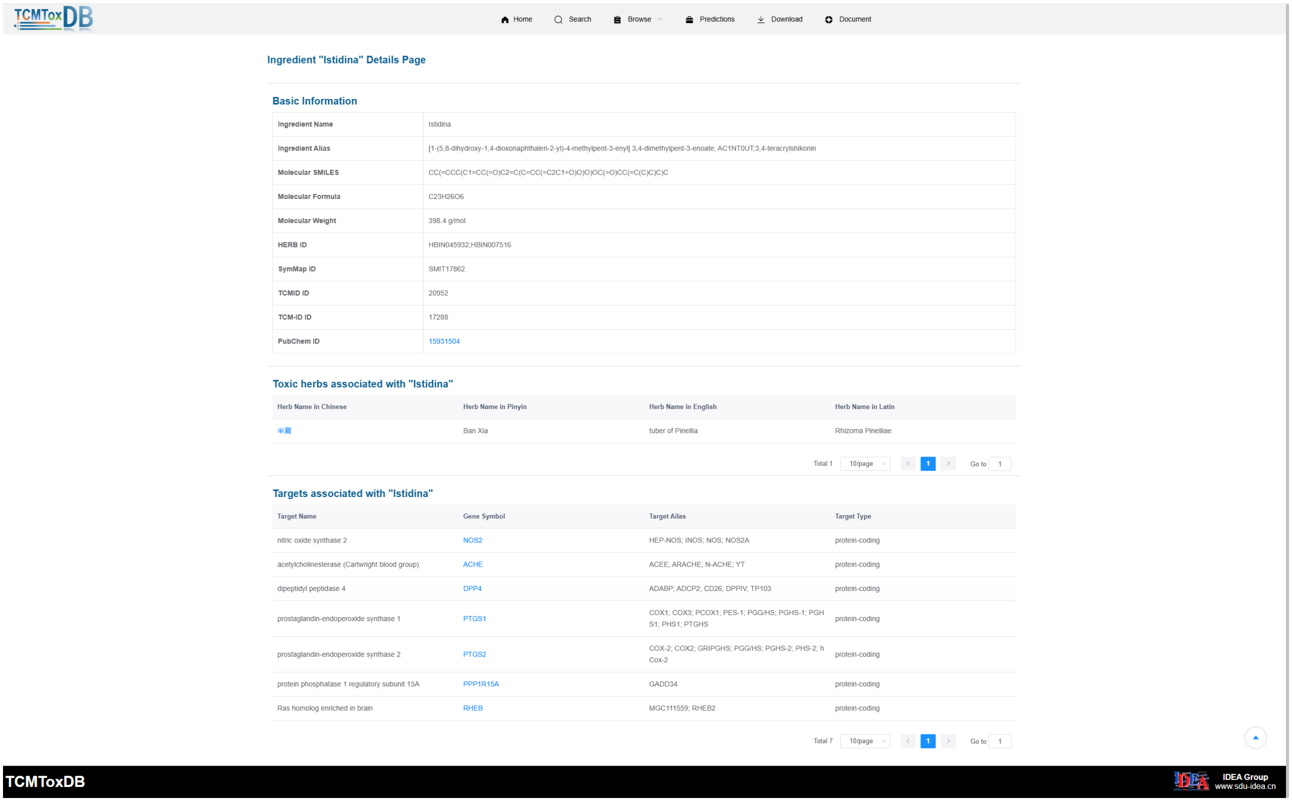

Figure S7. Page "Detail of Istidina". The page displays the basic information of the Istidina, toxic herb associated with Istidina, and targets associated with Istidina.

model for small molecule-protein interaction. In the input field, we may choose to enter either the name 'Istidina' or its SMILES representation to initiate the search. Figure S11 displays the results of searching for Istidina using its SMILES representation.

The system will generate a list of predicted targets for Istidina (as shown in Figure S12). The user can then cross-reference this list with the results from the herb-level prediction.

Downloading data

Beyond the specific prediction results, a dedicated "Download" page on TCMToxDB permits the extraction of data from the database. Downloadable content includes basic information on all formulas, herbs, ingredients, and targets (Figure S13).

Conclusion of Case Study

This case study demonstrates that TCMToxDB is more than a simple data repository; it is an integrated and intelligent research platform. In a logical, step-by-step workflow, a user can move from a broad query about a formula or herb to a specific toxicological mechanism at the molecular level. By bridging TCM knowledge with modern bioinformatics and predictive modeling, TCMToxDB empowers researchers to efficiently investigate the safety of traditional Chinese medicines, ultimately facilitating their modernization and safer clinical application.

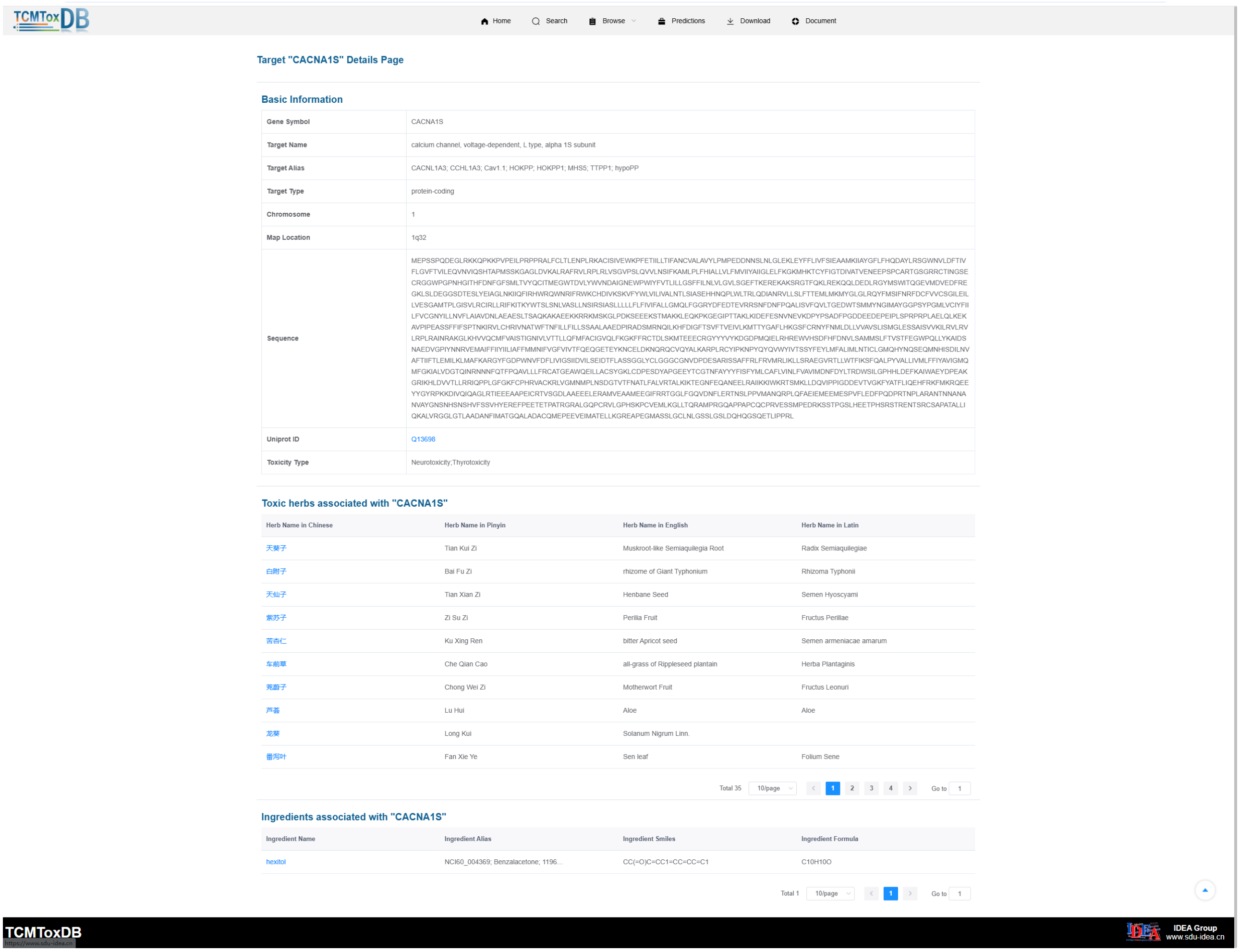

Figure S8. Page “Detail of CACNA1S”. The page displays the basic information of the CACNA1S, toxic herbs associated with CACNA1S, and ingredients associated with CACNA1S.

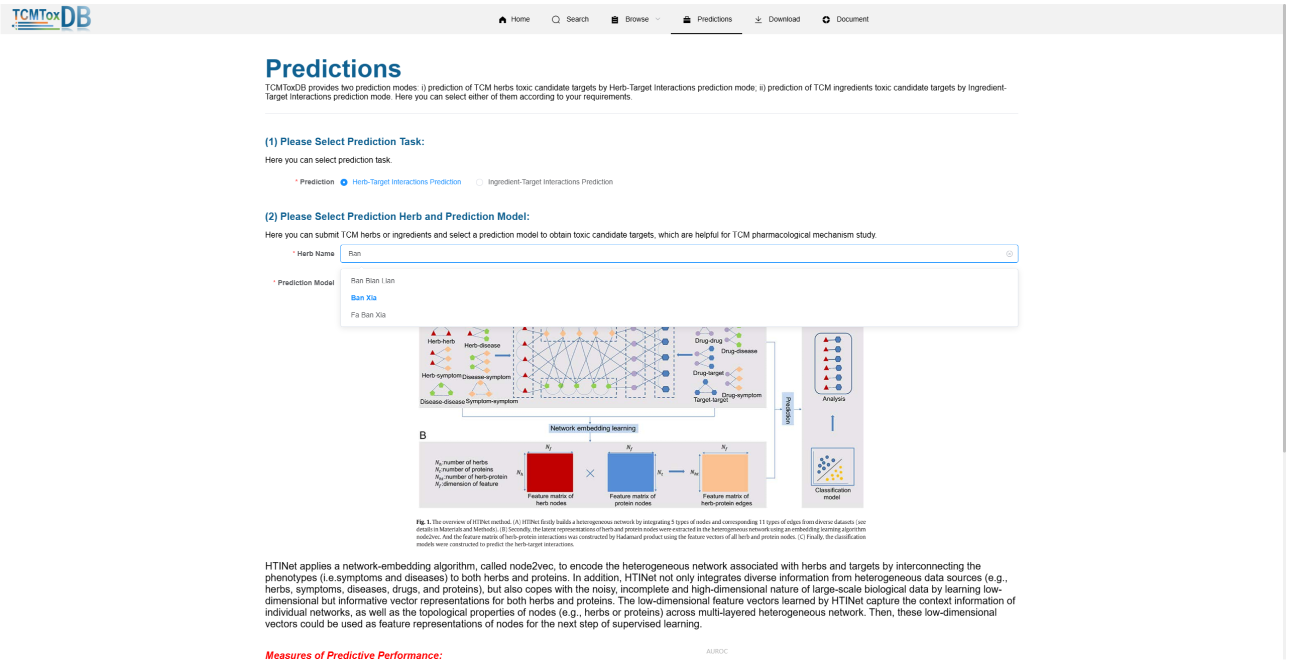

Figure S9. Page "Prediction" Section "Herb-Target Interactions Prediction". The figure shows the results when searching for the target herb using "Ban" as the keyword, with Ban Xia appearing in the auto-suggest dropdown menu.

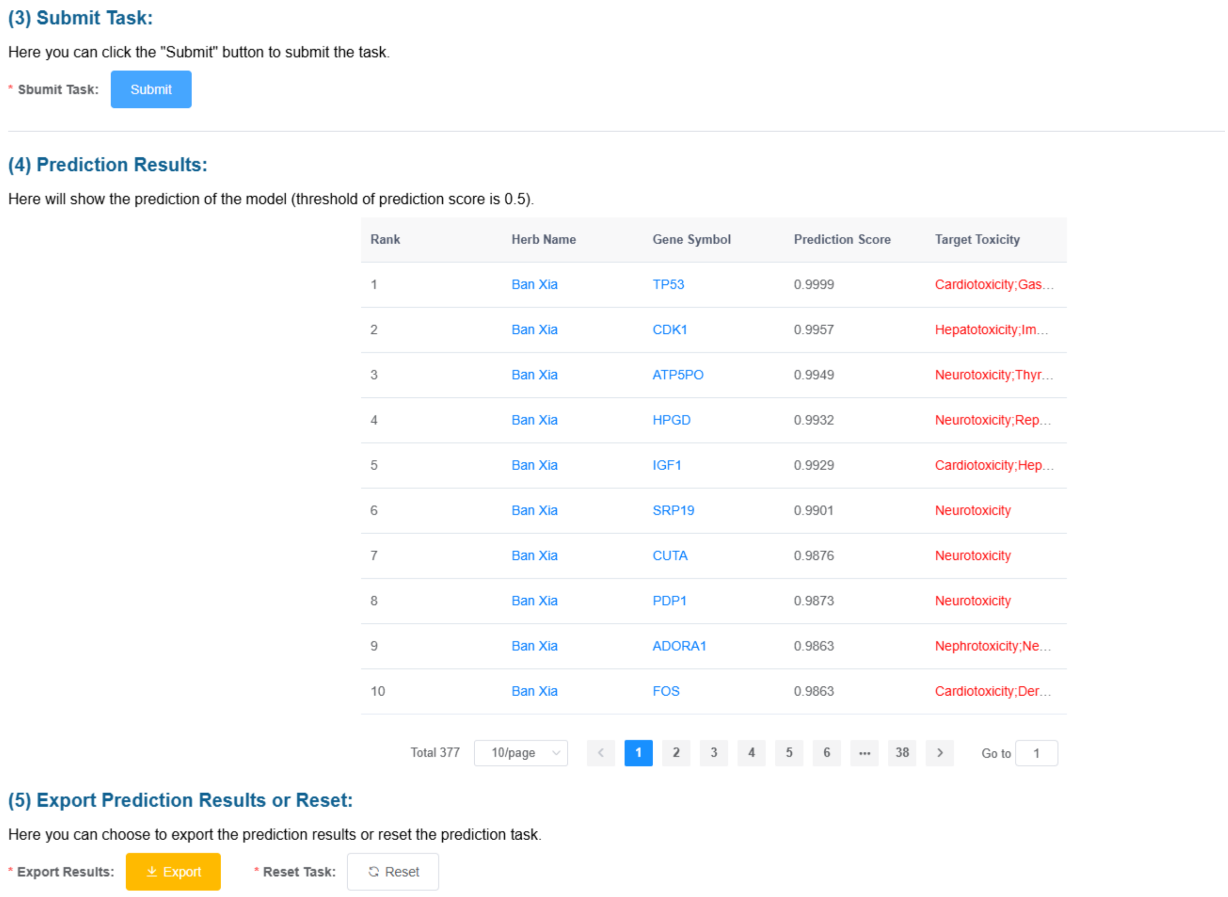

Figure S10. Page "Prediction" Section "Herb-Target Interactions Prediction". The figure displays the results of predicting putative toxic targets for Ban Xia using HTINet.

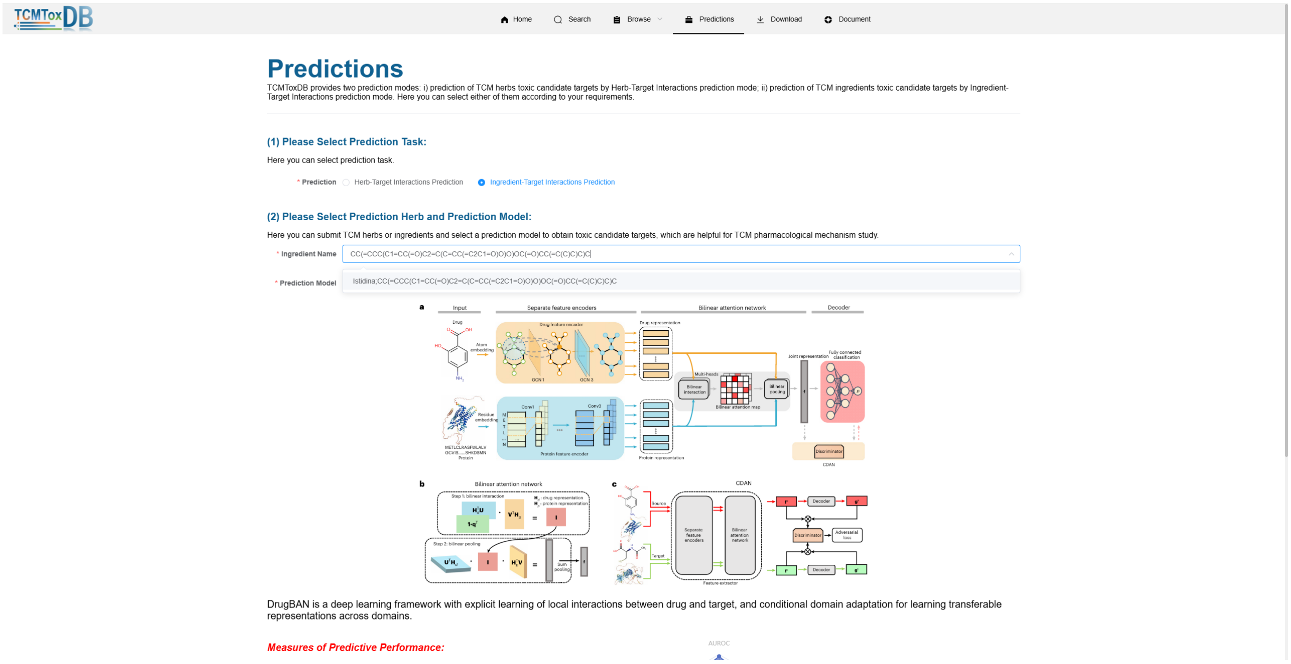

Figure S11. Page "Prediction" Section "Ingredient-Target Interactions Prediction". The figure shows the results when searching for Istidina using its SMILES representation as the keyword.

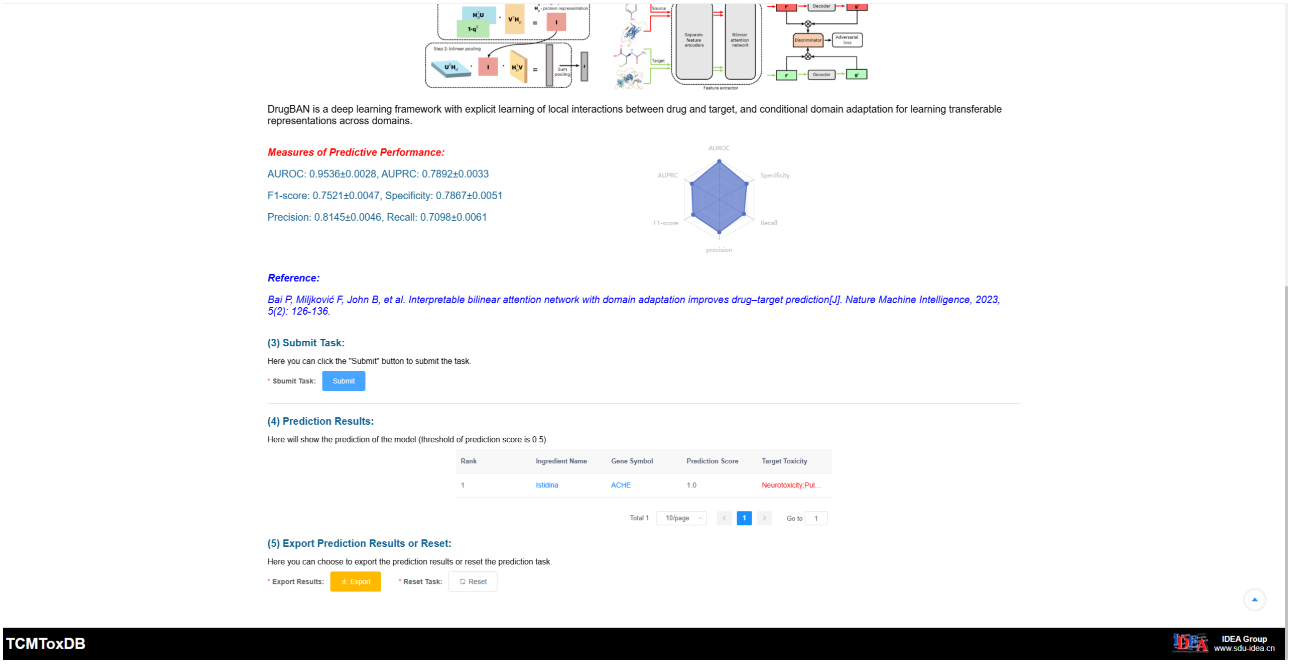

Figure S12. Page "Prediction" Section "Ingredient-Target Interactions Prediction". The figure shows the results of predicting putative toxic targets for Istidina using DrugBAN.

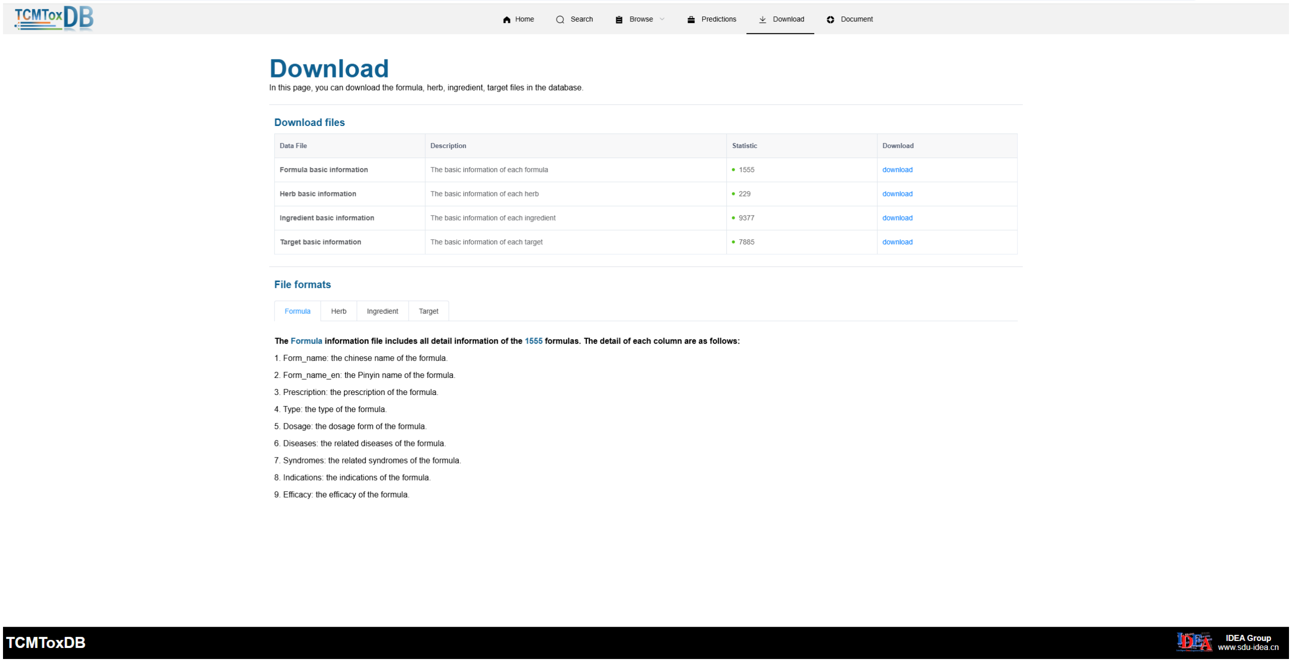

Figure S13. Page "Download". Downloadable content includes basic information on all formulas, herbs, ingredients, and targets.

Table S1. Scale statistics of databases related to toxicity study of TCMs.

Database	Formulas	Herbs	Ingredients	Targets	Relations (reported)
CTD [1]	N/A	N/A	15,279 (unique chemicals)	58,062 (unique genes)	3,279,901 chemical–gene interactions (curated); also reports chemical–disease and gene–disease associations (CTD Data Status, Feb 2026).
LiverTox [2]	N/A	N/A	N/A	N/A	>1,600 drug/agent records (monographs); LiverTox Database Updated: 26 Feb 2026.
Hepatox [3]	11	76	N/A	N/A	DILI knowledgebase; counts are from Hepatox index pages (Chinese patent drugs / Chinese herbs), and it does not provide structured ingredient–target interaction totals.
SIDER [4]	N/A	N/A	1430 (drugs)	N/A	139,756 drug–side effect pairs; 5,868 side effects (SIDER 4.1, released 2015-10-21).
HIM [5]	N/A	673	361	N/A	In vivo metabolism-related entries: 1,104 metabolites (ingredient/metabolite related; target relations NR).
TCMID [6]	46,929	8,159	43,413	17,603	Targets are derived from TCMID 1.0 reported targets (17,521) plus 82 new targets reported in TCMID 2.0; relation totals not uniformly reported.
TCM-ID [7]	7,443	2,751 (prescription components)	7,375	768	Targets include 463 human proteins and 305 pathogenic microbes; relation totals not reported on the statistics page.
TCMSP [8]	N/A	499	29,384	3,311	Provides ingredient–target and target–disease links; core relation/edge totals not uniformly reported (NR).
SymMap [9]	N/A	499	19,595	4,302	Reported examples: Herb–Syndrome 6,338; Herb–Ingredient 48,372; Ingredient–Target 29,370; Target–Disease 7,256 (others NR).
TCMBank [10]	N/A	9,192	61,966	15,179	Provides six pairwise relations (Herb–Ingredient, Herb–Target, Herb–Disease, Ingredient–Target, Ingredient–Disease, Target–Disease.); per-relation edge totals not uniformly reported (NR).
ETCM [11]	48,442	2,079	38,298	1,040	Core relation/edge totals not uniformly reported (NR).
HERB [12]	NR	7,263	49,258	12,933	Provides six pairwise relationships among entities; per-relation edge totals are not explicitly reported in the paper.
HIT [13]	N/A	1,254	1,237	2,208	Compound–target activity pairs: 10,031 (literature-evidence based).
TCMToxDB	1,555	229	9,377	7,885	Formula–Herb 5,119; Herb–Ingredient 16,603; Herb–Target 135,433; Ingredient–Target 65,332.

Notes: Counts are reported as stated in the corresponding database publications/websites when available. For the last column, relation counts are provided only when explicitly reported by the source; otherwise we summarize relation types and relevant notes for scope alignment. N/A: not applicable to the database schema; NR: not explicitly reported.

Table S2. Average association density across databases (derived from Table S1).

Database	Avg. ingredients per herb	Avg. targets per herb	Avg. targets per ingredient
TCMToxDB	40.95	34.43	0.84
TCMBank [10]	6.74	1.65	0.24
HERB [12]	6.78	1.78	0.26
TCMID [6]	5.32	2.16	0.41
TCM-ID [7]	2.68	0.28	0.10
TCMSP [8]	58.89	6.63	0.11
SymMap [9]	39.27	8.62	0.22
ETCM [11]	18.42	0.50	0.03
HIM [5]	0.54	N/A	N/A
HIT [13]	0.99	1.76	1.78
CTD [1]	N/A	N/A	N/A
LiverTox [2]	N/A	N/A	N/A
Hepatox [3]	N/A	N/A	N/A
SIDER [4]	N/A	N/A	N/A

Notes: Values are computed as Ingredients/Herbs, Targets/Herbs, and Targets/Ingredients where applicable (TCM-ID uses "prescription components" as the herb-level denominator as reported). N/A indicates not applicable to the database schema.

Database scope and interpretation of Table S1 and Table S2

Although several compared resources report substantially larger numbers of formulas/herbs/ingredients/targets, these databases are generally designed as broad pharmacology-oriented repositories that cover a wide spectrum of entities and relations beyond toxicity. In contrast, TCMToxDB is intentionally curated as a toxicity-focused resource, and thus the reported scale reflects a toxicity-specific subset rather than the entire TCM space. For example, all 229 herbs in TCMToxDB are toxic herbs, and only 217 out of 1,555 formulas are toxic formulas. In addition, our toxicity-oriented extraction contains 229 toxic herbs, 1,033 ingredients, and 2,047 toxic targets, together with curated toxic herb–target and ingredient–target relations. Therefore, the apparent difference in scale in Table S1 is primarily driven by different scopes and curation goals, rather than incomplete data collection. TCMToxDB complements existing resources by providing toxicity-centered, evidence-traceable records and dedicated toxicity-target prediction utilities. Notably, when evaluated by the average number of ingredients and targets per herb, as well as targets per ingredient (Table S2), TCMToxDB exhibits a significantly higher density of toxicity-related associations: each toxic herb is linked to an average of 41 ingredients and 34 targets, and each toxic ingredient is linked to 0.84 targets. This density is substantially higher than most broad pharmacology databases, indicating that TCMToxDB provides a more concentrated and detailed annotation of toxicity-relevant herb–ingredient–target networks, rather than a sparse, general-purpose collection.

Selected prediction models and their adoption in TCMToxDB

In TCMToxDB, toxicity-target analysis is conducted mainly at the ingredient–target level. To better characterize the pharmacological mechanisms of herbs, predictions are performed at both herb–target and ingredient–target levels. Each herb is first mapped to its curated ingredients, and ingredient–target prediction is then performed using standardized compound structures (e.g., canonical SMILES / molecular graphs) and target representations (e.g., protein sequences and normalized identifiers). The five models integrated in TCMToxDB were selected as representative baselines because they are widely used and recognized by the community, have reproducible implementations, and require practical and standardized inputs suitable for stable web deployment. Among these models, HTINet and HGNA-HTI are specialized herb–target interaction (HTI) prediction models, while DeepDTA, GraphDTA, and DrugBAN are classic compound–target interaction (DTI) prediction models.

HTINet.[14] HTINet is a herb–target interaction prediction framework that integrates multiple sources of evidence at the network level to infer potential herb–target associations. It is designed to enrich herb–target knowledge beyond limited curated herb–compound–target links by exploiting additional relational signals, and it outputs ranked candidate targets for herbs based on the integrated network inference. HTINet was chosen for its early development and representative role in herb–target interaction prediction, as it is specifically designed for holistic herb-level inference and compatible with network-based inputs that do not require detailed molecular structures, making it well suited for TCM research and reproducible web deployment.

HGNA-HTI.[15] HGNA-HTI models herb–target interaction prediction on a heterogeneous graph, where different node/edge types encode multiple biomedical relations. It employs an attention-based mechanism to aggregate information from heterogeneous neighbors and relation types, thereby learning herb and target representations suitable for ranking potential HTIs. As a heterogeneous graph attention model applied to herb–target interaction prediction, HGNA-HTI can model complex biological relationships; its attention mechanism may contribute to better feature representation and interpretability, and it supports standardized graph inputs that can facilitate stable deployment in the database.

DeepDTA.[16] DeepDTA is an end-to-end deep learning model for drug–target binding affinity prediction that encodes drug and protein inputs using 1D representations (e.g., strings/sequences) and learns the interaction signal through neural feature extraction. It provides a standard sequence-based DTI baseline with relatively simple inputs. DeepDTA was selected due to its wide adoption as a foundational sequence-based DTI model; it uses simple and universal inputs including SMILES and protein sequences, which are highly consistent with the standardized data format in TCMToxDB, and its lightweight architecture supports efficient and stable large-scale prediction.

GraphDTA.[17] GraphDTA represents compounds as molecular graphs and learns graph neural network embeddings for affinity/interaction prediction with protein information, leveraging the structural inductive bias of molecular graphs. It is a representative graph-based DTI baseline widely adopted in the community. GraphDTA is widely recognized as one of the most representative graph-based DTI methods, which can effectively capture molecular structural features critical for ingredient–target analysis; it supports standardized molecular graph inputs and tends to achieve a good balance between accuracy and efficiency, making it appropriate for large-scale web-based prediction in TCMToxDB.

DrugBAN.[18] DrugBAN learns fine-grained drug–target interactions using bilinear attention, highlighting local interaction patterns and improving generalization to novel drug–target pairs. It is a representative attention-based DTI model that is also useful for interpretability through attention-based interaction patterns. DrugBAN was selected for its strong predictive performance as a representative attention-based DTI model, which enables fine-grained interaction modeling and favorable generalization ability; its bilinear attention mechanism contributes to interpretability, and it is compatible with the standardized input pipeline of TCMToxDB.

We emphasize that these deployed predictors support both herb–target and ingredient–target inference and should not be interpreted as modeling holistic herb-level pharmacology without considering formula context and other clinical factors. To ensure consistent use across models, TCMToxDB standardizes ingredient identifiers/structures and target identifiers, unifies input and output formats, and reports prediction scores as ranked candidate targets with corresponding confidence scores.

References

1. Davis AP, Grondin CJ, Johnson RJ, et al. Comparative Toxicogenomics Database (CTD): Update 2021. *Nucleic Acids Res.* 2021;49(D1):D1138–D1143. https://doi.org/10.1093/nar/gkaa891.
2. Hoofnagle JH. LiverTox: A Website on Drug-Induced Liver Injury. In: *Drug-Induced Liver Disease.* 2013:725–732. https://doi.org/10.1002/hep.26175.
3. Mao Y. HepaTox: A Professional Network Platform to Promote Clinical and Translational Research on Drug-Induced Liver Injury in China. *Chinese Hepatology.* 2014;8(1):575–576. https://doi.org/10.3969/j.issn.1008-1704.2014.08.005.
4. Kuhn M, Letunic I, Jensen LJ, et al. The SIDER Database of Drugs and Side Effects. *Nucleic Acids Res.* 2016;44(D1):D1075–D1079. https://doi.org/10.1093/nar/gkv1075.
5. Kang H, Tang K, Liu Q, et al. HIM-Herbal Ingredients In-Vivo Metabolism Database. *J Cheminform.* 2013;5:1–6. https://doi.org/10.1186/1758-2946-5-28.
6. Huang L, Xie D, Yu Y, et al. TCMID 2.0: A Comprehensive Resource for TCM. *Nucleic Acids Res.* 2018;46(D1):D1117–D1120. https://doi.org/10.1093/nar/gkx1028.
7. Chen X, Zhou H, Liu YB, et al. Database of Traditional Chinese Medicine and Its Application to Studies of Mechanism and to Prescription Validation. *Br J Pharmacol.* 2006;149(8):1092–1103. https://doi.org/10.1038/sj.bjp.0706945.
8. Ru J, Li P, Wang J, et al. TCMSP: A Database of Systems Pharmacology for Drug Discovery from Herbal Medicines. *J Cheminform.* 2014;6:1–6. https://doi.org/10.1186/1758-2946-6-13.
9. Wu Y, Zhang F, Yang K, et al. SymMap: An Integrative Database of Traditional Chinese Medicine Enhanced by Symptom Mapping. *Nucleic Acids Res.* 2019;47(D1):D1110–D1117. https://doi.org/10.1093/nar/gky1021.
10. Lv Q, Chen G, He H, et al. TCMBank-The Largest TCM Database Provides Deep Learning-Based Chinese-Western Medicine Exclusion Prediction. *Signal Transduct Target Ther.* 2023;8(1):127. https://doi.org/10.1038/s41392-023-01339-1.
11. Zhang Y, Li X, Shi Y, et al. ETCM v2.0: An Update with Comprehensive Resource and Rich Annotations for Traditional Chinese Medicine. *Acta Pharm Sin B.* 2023;13(6):2559–2571. https://doi.org/10.1016/j.apsb.2023.03.012.
12. Fang S, Dong L, Liu L, et al. HERB: A High-Throughput Experiment-and Reference-Guided Database of Traditional Chinese Medicine. *Nucleic Acids Res.* 2021;49(D1):D1197–D1206. https://doi.org/10.1093/nar/gkaa1063.
13. Yan D, Zheng G, Wang C, et al. HIT 2.0: An Enhanced Platform for Herbal Ingredients' Targets. *Nucleic Acids Res.* 2022;50(D1):D1238–D1243. https://doi.org/10.1093/nar/gkab1011.
14. Wang N, Li P, Hu X, et al. Herb Target Prediction Based on Representation Learning of Symptom Related Heterogeneous Network. *Comput Struct Biotechnol J.* 2019;17:282–290. https://doi.org/10.1016/j.csbj.2019.02.002.
15. Zhao W, Wu H, He J. HGNA-HTI: Heterogeneous Graph Neural Network with Attention Mechanism for Prediction of Herb-Target Interactions. In: *IEEE Int. Conf. Bioinformatics Biomed. (BIBM).* 2021:3949–3956. https://doi.org/10.1109/BIBM52615.2021.9669308.
16. Öztürk H, Özgür A, Ozkirimli E. DeepDTA: Deep Drug–Target Binding Affinity Prediction. *Bioinformatics.* 2018;34(17):i821–i829. https://doi.org/10.1093/bioinformatics/bty593.
17. Nguyen T, Le H, Quinn TP, et al. GraphDTA: Predicting Drug–Target Binding Affinity with Graph Neural Networks. *Bioinformatics.* 2021;37(8):1140–1147. https://doi.org/10.1093/bioinformatics/btaa921.
18. Bai P, Miljković F, John B, et al. Interpretable Bilinear Attention Network with Domain Adaptation Improves Drug–Target Prediction. *Nat Mach Intell.* 2023;5(2):126–136. https://doi.org/10.1038/s42256-022-00605-1.
